# Association Between Water Intake and Mortality Risk—Evidence From a National Prospective Study

**DOI:** 10.3389/fnut.2022.822119

**Published:** 2022-04-12

**Authors:** Hao-long Zhou, Mu-hong Wei, Yuan Cui, Dong-sheng Di, Wen-jing Song, Ru-yi Zhang, Jun-an Liu, Qi Wang

**Affiliations:** ^1^MOE Key Lab of Environment and Health, Department of Epidemiology and Biostatistics, School of Public Health, Tongji Medical College, Huazhong University of Science and Technology, Wuhan, China; ^2^Department of Social Medicine and Health Management, School of Public Health, Tongji Medical College, Huazhong University of Science and Technology, Wuhan, China

**Keywords:** all-cause mortality, cancer/malignant neoplasms mortality, heart disease mortality, dose–response relationship, National Health and Nutrition Examination Survey, water intake

## Abstract

**Background:**

Few studies have explored the association between water intake and mortality risk, and the findings were inconsistent.

**Objective:**

This study aimed to explore the water intake–mortality association, utilizing the data from the National Health and Nutrition Examination Survey (NHANES) and the 2015 public-linked mortality files released by the National Center for Health Statistics.

**Methods:**

We used the diet- and mortality-linked data of a total of 35,463 adults (17,234 men) aged ≥20 years in the NHANESs 1999–2014 to perform a prospective study. The multivariate-adjusted Cox proportional hazards model was used to explore the associations of the amount of water intake (expressed by total water, plain water, beverage, and food water) and water intake proportion (expressed by the percentage of each kind of water) with mortality risks due to all causes, malignant neoplasms/cancer, and heart disease. The restricted cubic spline plots were adopted to clarify the dose–response relationships among them.

**Results:**

With a median of 88 months (interquartile range: 49–136 months) follow-up, a total of 4,915 all-cause deaths occurred, including 1,073 and 861 deaths from malignant neoplasms/cancer and heart disease, respectively. The amount of water intake in either type was negatively associated with all-cause mortality risk. Additionally, the negative linear dose–response relationships of water intake and all-cause mortality risk were found for all types of water except for food water, which followed a non-linear pattern. Similarly, compared to the lowest quartile (beverage water intake: <676 g/day; food water intake: <532 g/day), beverage and food water intakes in the range of 1,033–1,524 and 1,612–3,802 g/day were associated with decreased malignant neoplasms/cancer mortality risk. A U-shaped dose–response relationship was found for beverage water intake and malignant neoplasms/cancer mortality risk and a negative linear dose–response relationship was found for food water intake and malignant neoplasms/cancer mortality risk. Coffee and/or tea consumption was/were negatively associated with mortality risks due to all causes and malignant neoplasms/cancer. No significant associations of water intake proportion and mortality risks were found.

**Conclusion:**

Our findings demonstrated that higher water intake is associated with lower mortality risks among the United States population.

## Introduction

Water is the major component of the human body, accounting for approximately 50–55 and 60% of the bodyweight of women and men, respectively (1). It is recognized as a vital nutrient and plays various roles, such as solvent, reaction medium, reactant, carrier, lubricant, and shock absorber (2) in the maintenance of normal life activities. Generally, water can be ingested as moisture in food and beverage or comes from metabolism, but often it is not enough to fulfill people’s needs (2). Consequently, we need to consume extra plain water for replenishment. Based on the “Daily Water Intake Among U.S. Men and Women, 2009–2012” (3), American men and women consume an average of 3.46 and 2.75 L of water from all the food and liquids per day, and plain water contributes 30 and 34% of the total water intake in men and women, respectively. The water requirements recommended by the European Food Safety Authority are 2.5 and 2 L/day for men and women (1). However, it is impossible to give an individualized optimal value of water intake due to individual variability, as well as the different states of physical activity and climatic conditions (4).

Previous studies have explored the associations among tea, milk, alcohol, coffee consumption, and multiple health-related outcomes (5–8). However, few studies have focused on the associations of water intake with mortality risks or other chronic diseases. Animal experiment studies found that inadequate water intake was the leading cause of mortality induced by post-filament middle cerebral artery occlusion (9). Kant et al. concluded that different sources of water intake and total water intake were not associated with all-cause mortality risk in men; however, women in the highest category of total water intake were associated with higher all-cause mortality risk (10). Nevertheless, several studies pointed out that there were no significant associations between water intake and mortality risks due to rapid renal decline, cardiovascular disease, breast cancer, or ischemic heart disease (11–15). Inadequate water intake would affect normal organism metabolism and excessive water consumption may lead to hyponatremia (16), both of which are hazardous to health. The abovementioned studies did not describe the dose–response relationship between water intake and mortality risk. Furthermore, as far as we know, no study explored the associations of water intake proportion with mortality risks.

We conducted a prospective study to explore the associations of the amount and the proportion of water intake (expressed by total water, plain water, beverage, and food water) with mortality risks due to all causes, malignant neoplasms/cancer, and heart disease in United States adults. Considering differences in water requirements and consumption by different genders and the inconsistent water intake–mortality risk associations reported in men and women, the results were stratified by gender to explore the potential modification by gender.

## Materials and Methods

### Study Design and Participants

We conducted a prospective study using the dietary data from the National Health and Nutrition Examination Surveys (NHANESs) 1999–2014 and the mortality data from the 2015 public-linked mortality files released by the National Center for Health Statistics (NCHS). The NHANES is a program conducted by the NCHS of the Centers for Disease Control and Prevention (CDC), aiming to assess the health and nutritional status of adults and children in the United States. The NHANES was conducted using a complex multistage probability sampling design to select a representative sample of the civilian non-institutionalized household population. The protocol of NHANES was approved by the NCHS Research Ethics Review Board. Written consent was obtained from each participant.

Beginning in 1999, the NHANES became an annual survey and was on a 2-year data release cycle. The NHANES collected data of demographic, dietary, physical examination, laboratory detection, etc. We combined eight rounds of survey data from 1999 to 2014 in this prospective study. All respondents that participated in the NHANES were eligible for inclusion (*n* = 82,091). Participants were excluded in case (1) they only received an interview without physical examination (*n* = 3,573), (2) aged <20 years (*n* = 36,859), (3) who were pregnant (*n* = 1,344), (4) without available data on water intake or linkage mortality (*n* = 2,607), and (5) their values of water intake amount were outliers, i.e., above corresponding 97.5 percentiles (*n* = 2,245). Finally, a total of 35,463 adults including 17,234 men and 18,229 women were involved in our study. The flowchart for the inclusion and exclusion of participants is shown in Supplementary Figure 1.

### Exposure Ascertainment

Exposure variables included the amount of total water intake and water intake from plain water, beverage, and food, as well as water intake proportion. The trained and professional staff of NHANES collected the data on water intake, and then, the related data was reviewed and edited into an electronic system. Only the 1-day dietary intake data of each participant was released in the NHANESs 1999–2002, and the 2-day dietary intake data was released in the NHANESs 2003–2014, permitting the estimation of usual (long-run average) nutrient intakes in the United States population. The first-day data were collected in the mobile examination center, and the second-day data were collected by telephone 3–10 days later. Therefore, we used the average amount of water intake data from the 2 days for statistical analysis.

Beverage and food water intake data were collected through the 24-h recall in the NHANESs 1999--2014, while the amount of plain water consumption during the 24 h prior to the dietary interview was obtained by self-reporting and the 24-h recall in the NHANESs 1999--2014 and 2005--2014, respectively. The Automated Multiple Pass Method (AMPM), which was designed to collect the data of food intake efficiently and accurately for large-scale national surveys, was adopted in the 24-h dietary recall data collection. The AMPM was validated in a large study and demonstrated to be an effective method for collecting the accurate group food components and energy intake of adults.^1^ Plain water indicated plain tap water, water from a drinking fountain or water cooler, bottled water, and spring water. The NHANES provided data on the moisture contents of each kind of food and beverage reported in the dietary recall, as well as the summary data on the total moisture intake from food and beverages. The total beverage water intake was calculated by adding up the beverage moistures of all types, including milk and milk products, fruit juices, non-alcoholic beverages (coffee/coffee substitutes, tea, soft drinks/carbonated, and other non-alcoholic beverages), alcoholic beverages, and others using water as ingredients. Food water intake was calculated by subtracting the beverage water intake from the total moisture intake from food and beverage. The total water intake equaled the sum of water intake from plain water, beverage, and food. The proportion of water intake was defined as the corresponding proportions of the water types.

### Outcome Ascertainment

We used the 2015 public-linked mortality files released by NCHS. These files are available at the NHANES 1999–2014. The mortality status of each participant was identified primarily through probabilistic record matching with the National Death Index while the NCHS took other sources of mortality information into account, such as the social security administration, the centers for Medicare, Medicaid services, and the death certificate for determining vital status. These linked mortality files supplied information on the final mortality status, follow-up time, and underlying leading cause of death based on the International Classification of Disease-10-cause-of-death codes (ICD001-ICD010). Refer to the following website for details: https://www.cdc.gov/nchs/data-linkage/mortality-public.htm.

Up to now, the NCHS has only updated the public-use versions of the linked mortality files for the NHANES 1999–2014. The mortality follow-up period was defined as the death that occurred from the date of survey participation to December 31, 2015. The linked mortality files for the NHANESs 1999–2006 recorded a total of 10 kinds of leading causes of death, including “Disease of heart,” “Malignant neoplasms,” “Chronic lower respiratory,” “Accidents (unintentional injuries),” “Cerebrovascular diseases,” “Alzheimer’s disease,” “Diabetes mellitus,” “Influenza and pneumonia,” “Nephritis, nephrotic syndrome and nephrosis,” and “All other causes (residual).” Only three kinds of leading causes of death were recorded in the linked mortality files for the NHANESs 2007–2014, including “Disease of heart,” “Malignant neoplasms,” and “All other causes (residual).” Thus, we included the available data on the three shared leading causes of death—“All causes,” “heart disease,” and “malignant neoplasms”—as outcomes of interest in our NHANESs 1999–2014 study. All-cause death was defined as death due to any cause.

### Covariates

Covariates included gender (male, female), age (20–39, 40–59, and ≥60 years), race (Mexican American, Hispanics, non-Hispanic White, non-Hispanic Black, and others), body mass index (BMI) (<18.5,18.5–24.9, 25–29.9, ≥30 kg/m^2^), family income-to-poverty ratio (PIR), marital status (married/living with partner, widowed/separated/divorced, and never married), educational background (below high school, high school or equivalent, and above high school), smoking status (never, ever, and currently), drinking status (never, ever, and currently), leisure-time physical activity (sedentary, insufficient, moderate, and high), total energy intake, total protein intake, total carbohydrate intake, total lipid intake, total dietary fiber intake, and medical history of asthma, congestive heart failure, coronary heart disease, angina, heart attack, stroke, emphysema, thyroid disease, chronic bronchitis, liver condition, cancer or malignancy, high blood pressure, high blood cholesterol, diabetes, and failing kidneys.

The information on general demographic characteristics was obtained from interviews. The BMI was calculated by using weight (kg) divided by the square of height (m). The leisure-time physical activity was categorized into four groups based on metabolic equivalent (MET)-minutes per week: sedentary (MET = 0), insufficient (0 < MET ≤ 500), moderate (500 < MET ≤ 1,000), and high (MET > 1,000) (17). The information on total dietary energy, protein, carbohydrate, lipids, and fiber intake was obtained from a 24-h dietary recall. The information on medical history was collected by questionnaire survey. Considering excessive covariates adjusted in the model would affect the efficiency and power of models and co-linearity may exist in the medical history of different diseases, we adopted cluster analysis to categorize these medical histories into four clusters (Supplementary Table 1 and Supplementary Figure 2). Cluster 1 included medical histories of congestive heart failure, coronary heart disease, angina, heart attack, and stroke; cluster 2 included medical histories of high blood pressure, high blood cholesterol, and diabetes; cluster 3 included medical histories of asthma, emphysema, and bronchitis; and cluster 4 included medical histories of thyroid disease, liver condition, cancer/malignancy, and failing kidney. Each cluster was recorded as “Yes” when any medical history included was reported.

### Statistical Analysis

The SAS 9.4 software was used for statistical analysis. The analysis procedure for the complex and multistage probability sampling design was mainly referred to the NHANES analytic guidelines, and the sample weights were adopted in all analyses to ensure estimations could be representative for the United States population.^2^ General characteristics at the baselines were presented as mean (standard deviation), median (interquartile range), or number (percentage) where appropriate. The multivariate-adjusted Cox proportional hazards model was used to explore the associations of the amount of all kinds of water intake with mortality risks due to all causes, malignant neoplasms/cancer, and heart disease. Four kinds of water intake were categorized into four groups by respective quartiles: Q1 (<P_25_), Q2 (P_25_–P_50_), Q3 (P_50_–P_75_), and Q4 (>P_75_). Restricted cubic spline plots with three knots were used to clarify the dose–response relationship among them. Moreover, we adopted a multivariate-adjusted Cox proportional hazards model to explore the associations of the water intake proportion with mortality risks in the context of controlling for total water intake.

To determine the robustness of our results, we adjusted different covariates in the four Cox proportional hazards models. Covariates adjusted in model 1 included gender, age, race, marital status, educational background, BMI, and family PIR; covariates adjusted in model 2 included leisure-time physical activity, drinking status, smoking status, dietary energy, dietary protein, dietary carbohydrate, dietary lipids, and dietary fiber in addition to those adjusted in model 1; covariates adjusted in model 3 included medical histories of cluster 1–4 in addition to those adjusted in model 2; and for plain water, beverage, and food water, we added a model 4 with adjustment on the other kinds of water intake in addition to those adjusted in model 3 to ensure that all kinds of water intake were independent of each other. Similarly, the covariates adjusted in the restricted cubic spline plots were consistent with those adjusted in model 3 of the Cox proportional hazards model for the associations of total water intake with mortality risks, and the covariates adjusted in restricted cubic spline plots were consistent with those adjusted in model 4 of the Cox proportional hazards model for the associations of water intake from plain water, beverage water, and food water with mortality risks. All the above-mentioned analyses were stratified by gender to explore potential gender discrepancies. We also performed a subgroup analysis by beverage types to explore the associations of different types of beverage intake with mortality risks. Furthermore, different disease statuses might affect the amount of water intake. Sensitivity analyses were performed including only the generally healthy participants without a known medical history of any of the above-mentioned diseases, and those without a known medical history of malignancy/cancer and heart disease, respectively. All statistical analyses were two-sided and a *P*-value of <0.05 was considered statistically significant.

## Results

### General Characteristics

Our study included a total of 35,463 American adults and was split 50/50% between men and women. Nearly half of the subjects were non-Hispanic white (47%), and more than half of the subjects were married/cohabiting (60%). Only 10,010 subjects (29%) had a normal BMI (18.5–24.9 kg/m^2^) and most (70%) were physically inactive. In this prospective study of a median of 88 months (IQR: 49, 136) follow-up, a total of 4,915 all-cause deaths occurred, including 1,073 and 861 deaths from malignant neoplasms/cancer and heart disease, respectively. The medians of reported daily total water intake in all participants, men, and women were 2,970 (IQR: 2,151, 4,042), 3,149 (IQR: 2,310, 4,241), and 2,800 g/day (IQR: 2,019, 3,841), respectively. The proportions of water intake from plain water, beverage, and food were 26, 37, and 36% in all participants; the proportions were 24, 41, and 35% in men, and 28, 34, and 38% in women. In addition to plain water intake, the total water intake and the water intake from beverages and food in men were all higher than those in women (Table 1).

**TABLE 1:** General characteristics of participants at the baseline grouped by gender (NHANES 1999–2014).

Characteristics		Men (*n* = 17,234)	Women (*n* = 18,229)	Total (*n* = 35,463)
*n* (%)				
Age (years)	20–39	5,547 (32)	5,739 (31)	11,286 (32)
	40–59	5,402 (31)	5,986 (33)	11,388 (32)
	≥60	6,285 (36)	6,504 (36)	12,789 (36)
Race	Mexican American	3,127 (18)	3,238 (18)	6,365 (18)
	Other Hispanic	1,184 (7)	1,443 (8)	2,627 (7)
	Non-Hispanic White	8,152 (47)	8,475 (46)	16,627 (47)
	Non-Hispanic Black	3,627 (21)	3,880 (21)	7,507 (21)
	Others	1,144 (7)	1,193 (7)	2,337 (7)
Education	Under high school	5,070 (29)	5,067 (28)	10,137 (29)
	High school or equivalent	4,050 (24)	4,171 (23)	8,221 (23)
	Above high school	8,092 (47)	8,963 (49)	17,055 (48)
Marital status	Married/cohabiting	11,327 (66)	9,644 (54)	20,971 (60)
	Widowed/divorced/separated	2,646 (16)	5,445 (30)	8,091 (23)
	Never married	3,072 (18)	2,914 (16)	5,986 (17)
BMI (kg/m^2^)	<18.5	216 (1)	363 (2)	579 (2)
	18.5–24.9	4,681 (28)	5,329 (30)	10,010 (29)
	25.0–29.9	6,739 (40)	5,211 (29)	11,950 (34)
	≥30.0	5,292 (31)	7,009 (39)	12,301 (35)
Physical activity	Sedentary	0 (0)	0 (0)	0 (0)
	Insufficient	6,095 (66)	6,496 (74)	12,591 (70)
	Moderate	1,411 (15)	1,170 (13)	2,581 (14)
	High	1,704 (19)	1,096 (13)	2,800 (16)
Smoking status	Never	7,596 (44)	11,402 (63)	18,998 (54)
	Ever	5,503 (32)	3,602 (20)	9,105 (26)
	Current	4,119 (24)	3,212 (18)	7,331 (21)
Drinking status	Never	1,279 (44)	3,587 (50)	4,866 (49)
	Ever	974 (34)	1,792 (25)	2,766 (28)
	Current	648 (22)	1,726 (24)	2,374 (24)
Cluster 1 of medical history	Yes	2,333 (14)	1,828 (10)	4,161 (12)
	No	14,898 (86)	16,400 (90)	31,298 (88)
Cluster 2 of medical history	Yes	8,668 (50)	9,447 (52)	18,115 (51)
	No	8,565 (50)	8,780 (48)	17,345 (49)
Cluster 3 of medical history	Yes	2,487 (14)	3,487 (19)	5,974 (17)
	No	14,745 (86)	14,742 (81)	29,487 (83)
Cluster 4 of medical history	Yes	3,008 (17)	4,740 (26)	7,748 (22)
	No	14,224 (83)	13,488 (74)	27,712 (78)
No. of deaths due to all causes		2,738 (16)	2,177 (12)	4,915 (14)
No. of deaths due to cancer/malignant neoplasms		630 (4)	443 (2)	1,073 (3)
No. of deaths due to heart disease		537 (3)	324 (2)	861 (2)
Median (IQR)				
Family PIR		2 (1, 4)	2 (1, 4)	2 (1, 4)
Dietary energy (kcal/day)		2,183 (1,670, 2,794)	1,639 (1,276, 2,070)	1,871 (1,420, 2,439)
Dietary carbohydrate (g/day)		263 (197, 342)	206 (156, 265)	230 (172, 304)
Dietary protein (g/day)		85 (64, 111)	63 (48, 81)	73 (54, 96)
Dietary fat (g/day)		79 (56, 108)	60 (42, 81)	68 (48, 95)
Dietary fiber (g/day)		16 (11, 23)	13 (9, 18)	14 (10, 20)
Follow-up time (month)		87 (49, 135)	89 (51, 138)	88 (49, 136)
Total water intake (g/day)		3,149 (2,310, 4,241)	2,800 (2,019, 3,841)	2,970 (2,151, 4,042)
Q1		1,831 (1,483, 2,093)	1,577 (1,282, 1,812)	1,687 (1,365, 1,939)
Q2		2,731 (2,526, 2,941)	2,399 (2,214, 2,601)	2,560 (2,352, 2,762)
Q3		3,634 (3,392, 3,914)	3,264 (3,010, 3,531)	3,449 (3,199, 3,726)
Q4		5,205 (4,660, 6,037)	4,762 (4,227, 5,545)	4,981 (4,445, 5,792)
Plain water intake (g/day)		718 (281, 1,342)	781 (333, 1,406)	753 (311, 1,374)
Q1		15 (0, 178)	118 (0, 236)	81 (0, 218)
Q2		496 (385, 600)	541 (451, 665)	518 (430, 637)
Q3		978 (867, 1,170)	1,036 (918, 1,214)	1,003 (888, 1,185)
Q4		1,888 (1,598, 2,313)	1,888 (1,623, 2,318)	1,888 (1,617, 2,317)
Proportion of plain water intake in total water intake (%)		24 (12, 34)	28 (16, 38)	26 (14, 36)
Beverage water intake (g/day)		1,209 (806, 1,746)	885 (595, 1,293)	1,032 (676, 1,524)
Q1		579 (421, 695)	426 (319, 520)	484 (353, 587)
Q2		1,002 (908, 1,105)	739 (670, 808)	848 (762, 938)
Q3		1,442 (1,320, 1,584)	1,068 (974, 1,170)	1,240 (1,132, 1,368)
Q4		2,204 (1,945, 2,603)	1,675 (1,458, 2,063)	1,970 (1,713, 2,388)
Proportion of beverage water intake in total water intake (%)		41 (26, 57)	34 (21, 51)	37 (23,54)
Food water intake (g/day)		954 (566, 1,637)	891 (499, 1,590)	924 (532, 1,610)
Q1		396 (284, 484)	344 (251, 422)	365 (267, 451)
Q2		745 (654, 841)	673 (580, 773)	709 (617, 807)
Q3		1,235 (1,077, 1,421)	1,191 (1,035, 1,373)	1,212 (1,055, 1,396)
Q4		2,232 (1,886, 2,720)	2,149 (1,830, 2,607)	2,193 (1,860, 2,660)
Proportion of food water intake in total water intake (%)		35 (22, 45)	38 (23, 47)	36 (22, 46)

*NHANES, the National Health and Nutrition Examination Survey; BMI, body mass index; IQR, interquartile range; family PIR, ratio of family income to poverty.*

*Physical activity was categorized into four groups based on metabolic equivalent (MET)-minutes per week: sedentary (MET = 0), insufficient (0 < MET < 500), moderate (500 ≤ MET < 1,000), and high (MET ≥ 1,000). Cluster 1 of medical history included five diseases (congestive heart failure, coronary heart disease, angina, heart attack, and stroke); Cluster 2 of medical history included three diseases (hypertension, high blood cholesterol, and diabetes); Cluster 3 of medical history included three diseases (asthma, emphysema, and bronchitis); and Cluster 4 of medical history included four diseases (thyroid disease, liver conditions, weak/failure kidneys, and cancer/malignancy). Each cluster was reckoned as “Yes” when any medical history included was reported. Q1 represented the first quartile (<P_25_); Q2 represented the second quartile (P_25_–P_50_); Q3 represented the third quartile (P_50_–P_75_); and Q4 represented the highest quartile (>P_75_).*

### Water Intake and All-Cause Mortality Risk

**FIGURE 1:**
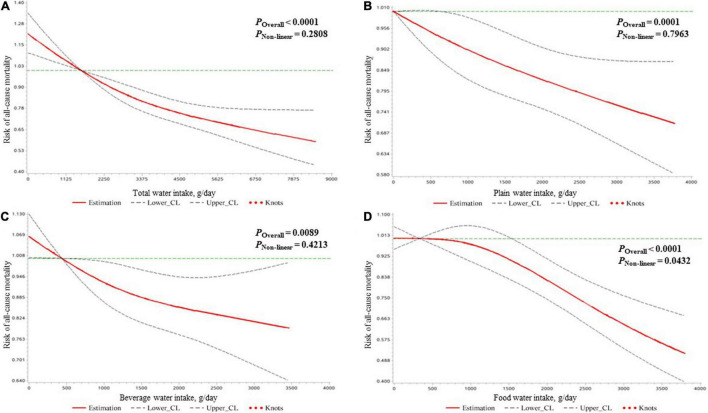
The dose–response relationships of the amount of water intake [expressed by total water **(A)**, plain water **(B)**, beverage water **(C)**, and food water **(D)**] with the mortality risk due to all causes in all participants clarified by restricted cubic spline plots.

In all participants, higher total water intake was associated with lower all-cause mortality risk after controlling for all covariates in model 3. The hazard ratios (HRs) (95% CIs) of all-cause mortality risk were 0.85 (0.76, 0.96), 0.77 (0.68, 0.88), and 0.77 (0.66, 0.90) in the second (2,151–2,970 g/day), third (2,971–4,042 g/day), and fourth (4,043–8,516 g/day) quartiles of total water intake compared to the first quartile (<2,151 g/day). Similarly, for beverage water intake, compared to the lowest quartile (<676 g/day), an intake >676 g/day was associated with lower all-cause mortality. As shown in model 4, the third quartile of plain water intake (755–1,374 g/day) and the fourth quartile of food water intake (1,612–3,802 g/day) was found to be independently associated with lower all-cause mortality risk compared to their lowest quartile (plain water intake: <311 g/day; food water intake: <532 g/day), respectively (Table 2). Using the restricted cubic spline method, the negative linear dose–response relationship of water intake with all-cause mortality risk was found for all types of water except food water, which followed a non-linear pattern (Figure 1).

**TABLE 2:** Associations of total water, plain water, beverage water, and food water intake with all-cause mortality risk in the NHANESs 1999–2014 participants.

Water intake	First quartile	Second quartile	Third quartile	Fourth quartile
**Model 1**				
Total water	1.00	**0.78 (0.70, 0.88)**	**0.69 (0.61, 0.78)**	**0.67 (0.58, 0.77)**
Plain water	1.00	0.92 (0.82, 1.05)	**0.78 (0.69, 0.88)**	**0.84 (0.74, 0.95)**
Beverage water	1.00	**0.81 (0.71, 0.92)**	**0.80 (0.70, 0.92)**	**0.70 (0.61, 0.80)**
Food water	1.00	0.94 (0.84, 1.05)	**0.79 (0.70, 0.88)**	**0.69 (0.59, 0.82)**
**Model 2**				
Total water	1.00	**0.85 (0.75, 0.96)**	**0.79 (0.69, 0.90)**	**0.79 (0.67, 0.93)**
Plain water	1.00	0.98 (0.87, 1.11)	**0.84 (0.74, 0.96)**	0.92 (0.81, 1.05)
Beverage water	1.00	**0.84 (0.73, 0.96)**	**0.85 (0.74, 0.98)**	**0.76 (0.65, 0.89)**
Food water	1.00	1.07 (0.95, 1.20)	0.93 (0.82, 1.07)	**0.82 (0.69, 0.97)**
**Model 3**				
Total water	1.00	**0.85 (0.76, 0.96)**	**0.77 (0.68, 0.88)**	**0.77 (0.66, 0.90)**
Plain water	1.00	0.94 (0.83, 1.07)	**0.81 (0.72, 0.92)**	**0.88 (0.77, 1.00)**
Beverage water	1.00	**0.84 (0.73, 0.97)**	0.88 (0.76, 1.02)	**0.78 (0.66, 0.92)**
Food water	1.00	1.07 (0.95, 1.21)	0.94 (0.83, 1.07)	**0.83 (0.70, 0.98)**
**Model 4**				
Total water	–			
Plain water	1.00	0.95 (0.84, 1.08)	**0.84 (0.74, 0.95)**	0.91 (0.79, 1.04)
Beverage water	1.00	**0.83 (0.72, 0.96)**	**0.85 (0.73, 0.99)**	**0.75 (0.63, 0.88)**
Food water	1.00	1.06 (0.94, 1.19)	0.94 (0.82, 1.08)	**0.84 (0.71, 0.98)**

*NHANES, National Health and Nutrition Examination Survey.*

*All estimates were calculated by multivariable Cox proportional hazards regression models, and results were expressed as hazard ratio (95% CI). Total water intake is the sum of water intake from plain water, beverage, and food. Four kinds of water intake were categorized into four groups according to respective quartiles. The covariates adjusted in model 1 included age, race, gender, education, marital status, the ratio of family income to poverty, and body mass index. The covariates adjusted in model 2 included leisure-time physical activity, dietary total energy, dietary protein, dietary carbohydrate, dietary total fat, dietary fiber, drinking, and smoking status in addition to those in model 1. The covariates adjusted in model 3 included clusters 1–4 of medical history in addition to those in model 2. The covariates adjusted in model 4 included the other kinds of water sources in addition to those in model 3. That is, for plain water, we included beverage water and food water as added covariates; for beverage water, we included plain water and food water as added covariates; and for food water, we included plain water and beverage water as added covariates.*

*The range of quartiles for total water intake was <2,515 (Q1), 2,151–2,970 (Q2), 2,971–4,042 (Q3), and 4,043–8,516 g/day (Q4). The range of quartiles for plain water intake was <311 (Q1), 311–754 (Q2), 755–1,374 (Q3), and 1,375–3,776 g/day (Q4). The range of quartiles for beverage water intake was <676 (Q1), 676–1,032 (Q2), 1,033–1,524 (Q3), and 1,525–3,464 g/day (Q4). The range of quartiles for food water intake was <532 (Q1), 532–924 (Q2), 925–1,611 (Q3), and 1,612–3,802 g/day (Q4). Boldness indicates a statistical significance.*

The analysis results of the associations of water intake with all-cause mortality risk stratified by gender are presented in Supplementary Tables 2, 3. No significant association between water intake and all-cause mortality risk was found in men (Supplementary Table 2). A negative linear dose–response relationship was found for total water intake and all-cause mortality risk, and a negative non-linear dose–response relationship was found for food water intake and all-cause mortality risk (Supplementary Figure 3). In women, higher total water intake in the second (2,019–2,800 g/day), third (2,801–3,841 g/day), and fourth (3,842–8,511 g/day) quartiles; the third quartile of plain water intake (782–1,406 g/day); the second (595–885 g/day) and fourth quartiles (1,294–3,464 g/day) of beverage water intake were found to be significantly associated with lower all-cause mortality risk compared to the lowest quartile (total water intake: <2,019 g/day; plain water intake: <333 g/day; beverage water intake: <595 g/day) (Supplementary Table 3). Negative linear dose–response relationships were found for water intake of all types and all-cause mortality risk (Supplementary Figure 4).

### Water Intake and Malignant Neoplasms/Cancer Mortality Risk

**FIGURE 2:**
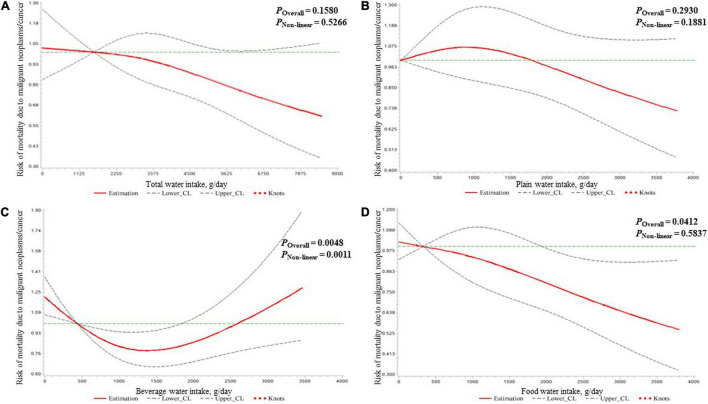
The dose–response relationships of the amount of water intake [expressed by total water **(A)**, plain water **(B)**, beverage water **(C)**, and food water **(D)**] with the mortality risk due to malignant neoplasms/cancer in all participants clarified by restricted cubic spline plots.

The results of the association of water intake and mortality risk due to malignant neoplasms/cancer in all participants are shown in Table 3. After adjusting for covariates, compared to the lowest quartile (beverage water intake: <676 g/day; food water intake: <532 g/day), the third quartile of beverage water intake (1,033–1,524 g/day) and the fourth quartile of food water intake (1,612–3,802 g/day) were associated with 35% (HR = 0.65, 95% CI: 0.48, 0.88) and 29% (HR = 0.71, 95% CI: 0.52, 0.98) reduced mortality risk due to malignant neoplasms/cancer, respectively. As shown in Figure 2, a U-shaped dose–response relationship was found for beverage water intake and malignant neoplasms/cancer mortality risk, and a negative linear dose–response relationship was found for food water intake and malignant neoplasms/cancer mortality risk.

**TABLE 3:** Associations of total water, plain water, beverage water, and food water intake with malignant neoplasms/cancer mortality risk in the NHANESs 1999–2014 participants.

Water intake	First quartile	Second quartile	Third quartile	Fourth quartile
**Model 1**				
Total water	1.00	0.94 (0.76, 1.15)	0.82 (0.63, 1.06)	**0.79 (0.63, 0.99)**
Plain water	1.00	0.81 (0.63, 1.03)	**0.77 (0.61, 0.99)**	0.80 (0.62, 1.03)
Beverage water	1.00	0.81 (0.61, 1.06)	**0.67 (0.50, 0.90)**	0.99 (0.75, 1.30)
Food water	1.00	0.90 (0.71, 1.14)	0.78 (0.60, 1.02)	**0.56 (0.42, 0.75)**
**Model 2**				
Total water	1.00	1.00 (0.81, 1.24)	0.92 (0.71, 1.20)	0.92 (0.72, 1.16)
Plain water	1.00	0.87 (0.67, 1.11)	0.85 (0.67, 1.08)	0.89 (0.69, 1.16)
Beverage water	1.00	0.79 (0.60, 1.05)	**0.66 (0.49, 0.88)**	0.95 (0.71, 1.28)
Food water	1.00	1.05 (0.82, 1.34)	0.96 (0.72, 1.28)	**0.70 (0.52, 0.96)**
**Model 3**				
Total water	1.00	1.00 (0.81, 1.24)	0.92 (0.70, 1.19)	0.91 (0.72, 1.15)
Plain water	1.00	0.85 (0.66, 1.09)	0.83 (0.65, 1.06)	0.88 (0.67, 1.14)
Beverage water	1.00	0.80 (0.60, 1.05)	**0.67 (0.50, 0.90)**	0.97 (0.72, 1.30)
Food water	1.00	1.05 (0.82, 1.35)	0.98 (0.74, 1.30)	**0.71 (0.53, 0.96)**
**Model 4**				
Total water	–			
Plain water	1.00	0.85 (0.65, 1.12)	0.87 (0.68, 1.13)	0.98 (0.73, 1.32)
Beverage water	1.00	0.78 (0.59, 1.04)	**0.65 (0.48, 0.88)**	0.93 (0.69, 1.26)
Food water	1.00	1.07 (0.83, 1.37)	1.04 (0.76, 1.41)	**0.71 (0.52, 0.98)**

*NHANES, National Health and Nutrition Examination Survey.*

*All estimates were calculated by multivariable Cox proportional hazards regression models, and results were expressed as hazard ratio (95% CI). Total water intake is the sum of water intake from plain water, beverage, and food. Four kinds of water intake were categorized into four groups according to respective quartiles. The covariates adjusted in model 1 included age, race, gender, education, marital status, the ratio of family income to poverty, and body mass index. The covariates adjusted in model 2 included leisure-time physical activity, dietary total energy, dietary protein, dietary carbohydrate, dietary total fat, dietary fiber, drinking, and smoking status in addition to those in model 1. The covariates adjusted in model 3 included clusters 1–4 of medical history in addition to those in model 2. The covariates adjusted in model 4 included the other kinds of water sources in addition to those in model 3. That is, for plain water, we included beverage water and food water as added covariates; for beverage water, we included plain water and food water as added covariates; and for food water, we included plain water and beverage water as added covariates.*

*The range of quartiles for total water intake was <2,515 (Q1), 2,151–2,970 (Q2), 2,971–4,042 (Q3), and 4,043–8,516 g/day (Q4). The range of quartiles for plain water intake was <311 (Q1), 311–754 (Q2), 755–1,374 (Q3), and 1,375–3,776 g/day (Q4). The range of quartiles for beverage water intake was <676 (Q1), 676–1,032 (Q2), 1,033–1,524 (Q3), and 1,525–3,464 g/day (Q4). The range of quartiles for food water intake was <532 (Q1), 532–924 (Q2), 925–1,611 (Q3), and 1,612–3,802 g/day (Q4). Boldness indicates a statistical significance.*

In men, no significant water intake–malignant neoplasms/cancer mortality risk association was found (Supplementary Table 4). However, we found a U-shaped dose–response relationship of beverage water intake with malignant neoplasms/cancer mortality risk (Supplementary Figure 5). In women, compared to the lowest quartile (<595 g/day), the fourth quartile (1,294–3,464 g/day) of beverage water intake was found to be independently and negatively associated with malignant neoplasms/cancer mortality risk, with an HR (95% CI) of 0.58 (0.36, 0.92) in model 4 (Supplementary Table 5). However, no significant dose-relationships of water intake with malignant neoplasms/cancer mortality risk were presented in Supplementary Figure 6.

### Water Intake and Heart Disease Mortality Risk

**FIGURE 3:**
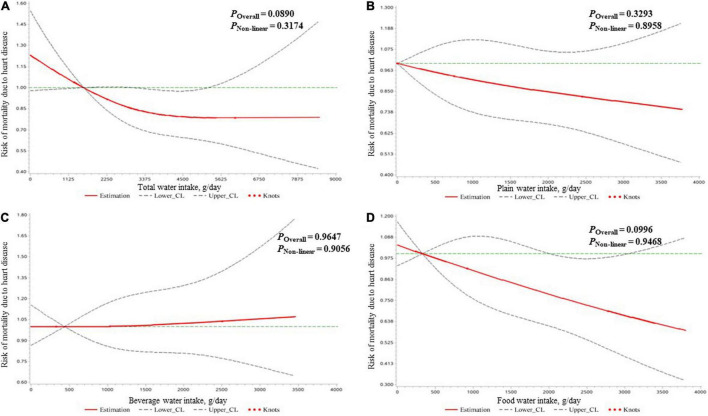
The dose–response relationships of the amount of water intake [expressed by total water **(A)**, plain water **(B)**, beverage water **(C)**, and food water **(D)**] with the mortality risk due to heart diseases in all participants clarified by restricted cubic spline plots.

As shown in Table 4, all kinds of water intake were not associated with heart disease mortality risk after adjusting all covariates. Also, no significant dose–response relationship of water intake and mortality risk due to heart disease was found (Figure 3).

**TABLE 4:** Associations of total water, plain water, beverage water, and food water intake with heart disease mortality risk in the NHANESs 1999–2014 participants.

Water intake	First quartile	Second quartile	Third quartile	Fourth quartile
**Model 1**				
Total water	1.00	0.76 (0.56, 1.03)	0.76 (0.56, 1.05)	**0.65 (0.46, 0.91)**
Plain water	1.00	0.98 (0.75, 1.29)	0.76 (0.56, 1.03)	0.90 (0.68, 1.18)
Beverage water	1.00	0.71 (0.51, 1.01)	**0.64 (0.47, 0.87)**	**0.59 (0.42, 0.83)**
Food water	1.00	0.91 (0.71, 1.18)	0.82 (0.62, 1.09)	0.78 (0.55, 1.12)
**Model 2**				
Total water	1.00	0.88 (0.64, 1.21)	0.95 (0.69, 1.31)	0.86 (0.60, 1.24)
Plain water	1.00	1.03 (0.78, 1.36)	0.81 (0.59, 1.11)	0.97 (0.72, 1.29)
Beverage water	1.00	0.79 (0.56, 1.12)	0.76 (0.56, 1.04)	0.79 (0.57, 1.09)
Food water	1.00	1.07 (0.81, 1.42)	1.01 (0.74, 1.38)	0.94 (0.66, 1.33)
**Model 3**				
Total water	1.00	0.88 (0.65, 1.19)	0.92 (0.67, 1.25)	0.82 (0.57, 1.18)
Plain water	1.00	0.96 (0.73, 1.26)	0.78 (0.57, 1.07)	0.90 (0.67, 1.21)
Beverage water	1.00	0.80 (0.57, 1.13)	0.81 (0.59, 1.12)	0.83 (0.60, 1.15)
Food water	1.00	1.04 (0.79, 1.39)	1.01 (0.74, 1.38)	0.94 (0.66, 1.33)
**Model 4**				
Total water	–			
Plain water	1.00	0.96 (0.72, 1.29)	0.78 (0.56, 1.07)	0.91 (0.66, 1.25)
Beverage water	1.00	0.79 (0.56, 1.11)	0.79 (0.57, 1.10)	0.80 (0.57, 1.13)
Food water	1.00	1.03 (0.78, 1.37)	1.02 (0.73, 1.42)	0.97 (0.68, 1.39)

*NHANES, National Health and Nutrition Examination Survey.*

*All estimates were calculated by multivariable Cox proportional hazards regression models, and results were expressed as hazard ratio (95% CI). Total water intake is the sum of water intake from plain water, beverage, and food. Four kinds of water intake were categorized into four groups according to respective quartiles. The covariates adjusted in model 1 included age, race, gender, education, marital status, the ratio of family income to poverty, and body mass index. Covariates adjusted in model 2 included leisure-time physical activity, dietary total energy, dietary protein, dietary carbohydrate, dietary total fat, dietary fiber, drinking, and smoking status in addition to those in model 1. The covariates adjusted in model 3 included clusters 1–4 of medical history in addition to those in model 2. The covariates adjusted in model 4 included the other kinds of water sources in addition to those in model 3. That is, for plain water intake, we included beverage water intake and food water intake as added covariates; for beverage water intake, we included plain water intake and food water intake as added covariates; and for food water intake, we included plain water intake and beverage water intake as added covariates.*

*The range of quartiles for total water intake was <2,515 (Q1), 2,151–2,970 (Q2), 2,971–4,042 (Q3), and 4,043–8,516 g/day (Q4). The range of quartiles for plain water intake was <311 (Q1), 311–754 (Q2), 755–1,374 (Q3), and 1,375–3,776 g/day (Q4). The range of quartiles for beverage water intake was <676 (Q1), 676–1,032 (Q2), 1,033–1,524 (Q3), and 1,525–3,464 g/day (Q4). The range of quartiles for food water intake was <532 (Q1), 532–924 (Q2), 925–1,611 (Q3), and 1,612–3,802 g/day (Q4). Boldness indicates a statistical significance.*

Similarly, this association was not significant in any gender (Supplementary Tables 6, 7). The gender-stratified restricted cubic spline plots illustrating the water intake (of all kinds studied) and heart disease mortality risk associations were also provided (Supplementary Figures 7, 8).

### Water Intake Proportions and Mortality Risks

The associations of water intake proportions and mortality risks were also explored in the adjustment of total water intake in addition to all other covariates, results of which are presented in Supplementary Table 8. No significant associations were found after controlling for all covariates.

### Different Kinds of Beverage Water Intake and Mortality Risks

As shown in Table 5, compared to those in the lowest quartile (coffee intake: <251 g/day; tea intake: <296 g/day; non-alcoholic beverage intake: <222 g/day), the participants in the second (251–390 g/day) and fourth (619–1,250 g/day) quartiles of coffee/coffee substitutes intake, and the second quartile of tea intake (296–463 g/day) were found to have lower all-cause mortality risk; the participants in the second quartile of coffee/coffee substitutes intake (251–390 g/day), and the second (296–463 g/day) and third (464–767 g/day) quartiles of tea intake were found to have lower malignant neoplasms/cancer mortality risk; the participants in the second quartile of other non-alcoholic beverages intake (222–375 g/day) had lower heart disease mortality risk. Surprisingly, compared to those in the lowest quartile (0–179 g/day), the participants in the third quartile of alcoholic beverages (386–911 g/day) water intake was associated with lower all-cause mortality risk, with an HR (95% CI) of 0.68 (0.49, 0.94).

**TABLE 5:** Associations of different beverage water intake with mortality risks due to all causes, malignant neoplasms/cancer and heart disease in the NHANESs 1999–2014 participants.

Beverage categories	First quartile	Second quartile	Third quartile	Fourth quartile
**All-cause mortality risk**				
Milk/milk drinks	1.00	1.11 (0.97, 1.27)	1.01 (0.89, 1.16)	1.14 (0.96, 1.35)
Fruit juices	1.00	1.27 (1.05, 1.52)	1.15 (0.93, 1.42)	1.01 (0.82, 1.24)
Coffee/coffee substitutes	1.00	**0.83 (0.69, 0.99)**	0.90 (0.77, 1.05)	**0.82 (0.69, 0.97)**
Tea	1.00	**0.81 (0.67, 0.98)**	0.83 (0.67, 1.03)	0.80 (0.64, 1.00)
Soft drinks/carbonated	1.00	0.86 (0.73, 1.02)	0.88 (0.74, 1.05)	0.81 (0.66, 1.01)
Other non-alcoholic beverages	1.00	0.89 (0.70, 1.14)	0.85 (0.66, 1.11)	1.00 (0.72, 1.38)
Alcoholic beverages	1.00	0.76 (0.57, 1.02)	**0.68 (0.49, 0.94)**	0.74 (0.54, 1.02)
**Malignant neoplasms/cancer mortality risk**				
Milk/milk drinks	1.00	0.95 (0.72, 1.25)	0.87 (0.65, 1.16)	1.07 (0.77, 1.47)
Fruit juices	1.00	1.09 (0.71, 1.66)	1.06 (0.66, 1.70)	0.98 (0.60, 1.59)
Coffee/coffee substitutes	1.00	**0.58 (0.42, 0.81)**	0.79 (0.58, 1.06)	0.91 (0.70, 1.17)
Tea	1.00	**0.62 (0.42, 0.94)**	**0.63 (0.42, 0.96)**	0.71 (0.47, 1.07)
Soft drinks/carbonated	1.00	0.93 (0.65, 1.33)	0.98 (0.67, 1.45)	0.89 (0.54, 1.45)
Other non-alcoholic beverages	1.00	1.14 (0.66, 1.97)	0.85 (0.46, 1.55)	0.72 (0.39, 1.33)
Alcoholic beverages	1.00	0.67 (0.38, 1.18)	0.69 (0.40, 1.20)	1.08 (0.59, 1.95)
**Heart disease mortality risk**				
Milk/milk drinks	1.00	0.96 (0.71, 1.30)	0.99 (0.69, 1.42)	1.13 (0.75, 1.70)
Fruit juices	1.00	1.04 (0.62, 1.75)	1.03 (0.67, 1.59)	0.66 (0.34, 1.26)
Coffee/coffee substitutes	1.00	1.03 (0.71, 1.50)	1.30 (0.91, 1.87)	0.93 (0.61, 1.43)
Tea	1.00	0.80 (0.52, 1.23)	0.83 (0.49, 1.40)	0.99 (0.63, 1.54)
Soft drinks/carbonated	1.00	0.81 (0.49, 1.35)	0.76 (0.47, 1.25)	0.89 (0.46, 1.70)
Other non-alcoholic beverages	1.00	**0.50 (0.27, 0.96)**	0.73 (0.33, 1.62)	1.23 (0.61, 2.51)
Alcoholic beverages	1.00	0.60 (0.28, 1.29)	0.57 (0.25, 1.25)	1.02 (0.51, 2.05)

*NHANES, National Health and Nutrition Examination Survey.*

*All estimates were calculated by multivariable Cox proportional hazards regression models, and results were expressed as hazard ratio (95% CI). Different kinds of beverage water intake were categorized into four groups according to respective quartiles. The covariates adjusted in the model included age, gender, race, education, marital status, the ratio of family income to poverty, body mass index, leisure-time physical activity, dietary total energy, dietary protein, dietary carbohydrate, dietary total fat, dietary fiber, drinking, and smoking status in clusters 1–4 of medical history, plain water, and food water intake.*

*The range of quartiles for milk/milk drinks intake was <120 (Q1), 120–219 (Q2), 220–361 (Q3), and 362–1,158 g/day (Q4). The range of quartiles for fruit juices intake was <112 (Q1), 112–208 (Q2), 209–308 (Q3), and 306–997 g/day (Q4). The range of quartiles for coffee/coffee substitutes intake was <251 (Q1), 251–390 (Q2), 391–618 (Q3), and 619–1,250 g/day (Q4). The range of quartiles for tea intake was <295 (Q1), 296–463 (Q2), 464–767 (Q3), and 768–1,644 g/day (Q4). The range of quartiles for soft drinks/carbonated intake was <331 (Q1), 331–463 (Q2), 464–746 (Q3), and 747–1,555 g/day (Q4). The range of quartiles for other non-alcoholic beverages intake was <222 (Q1), 222–375 (Q2), 376–586 (Q3), and 587–2,151 g/day (Q4). The range of quartiles for alcoholic beverages intake was <179 (Q1), 179–385 (Q2), 386–911 (Q3), and 912–2,293 g/day (Q4).*

*Boldness indicates a statistical significance.*

### Sensitivity Analyses

Overall, all significant associations of different kinds of water intake with mortality risks due to all causes, malignant neoplasms/cancer, and heart disease in the total population were also found in these subgroups, and the directions did not change (Supplementary Tables 9–11). The associations of food water intake with all-cause mortality risk and malignant neoplasms/cancer mortality risk changed to non-significant in the healthy population and those without cancer history (Supplementary Tables 9, 10).

## Discussion

In the large-scale prospective study of representative United States adults, we found that the amount of water intake in all the studied types was negatively associated with all-cause mortality risk, and water intakes from beverage and food in the range of 1,033–1,524 and 1,612–3,802 g/day were associated with reduced malignant neoplasms/cancer mortality risk compared to their lowest quartiles (beverage water intake: <676 g/day; food water intake: <532 g/day), respectively. A significantly negative linear dose–response relationship of water intake and all-cause mortality risk was found for all types of water except food water, which followed a non-linear pattern. A U-shaped dose–response relationship was also found for beverage water intake and malignant neoplasms/cancer mortality risk, and a negative linear dose–response relationship was found for food water intake and malignant neoplasms/cancer mortality risk. Most of these significant associations were seen in women but not in men, indicating that gender is an effective modifier of the water intake and mortality risk associations. Besides, water intake proportions were not associated with mortality risks. Compared to those in the lowest quartile (coffee intake: <251 g/day; tea intake: <296 g/day), the second quartiles of coffee intake (251–390 g/day) and tea intake (296–463 g/day) were found to be associated with decreased mortality risks due to all causes and malignant neoplasms/cancer. Surprisingly, alcoholic beverage intake of 386–911 g/day was also found to be associated with reduced all-cause mortality risk compared to that in the range of 0–179 g/day.

Traditionally, people were accustomed to linking more water intake with a healthier lifestyle, and the exhortation “drink at least eight glasses of water per day” was popular (18). Wu et al. utilized data from the third NHANES and reported that the subjects with a fluid intake ≥3.576 L/day had lower all-cause mortality risks than those with a fluid intake ≤2.147 L/day in the chronic kidney disease group (19). In a study that included 20,297 adults without heart disease, stroke, and diabetes, participants who drank five or more glasses of water per day were associated with a 54% decreased risk of fatal coronary heart disease compared with participants who drank two or fewer glasses of water per day (20). Also, several studies demonstrated that different sources of water intake were negatively associated with cancers, such as bladder cancer, breast cancer, and colorectal cancer (21–23). These studies were in line with our findings. However, inconsistent conclusions were also reported in several studies. A research aiming at the Australian population aged 49 years or older found no evidence for decreased mortality or improved kidney function with higher daily water intake (14). Kant et al. concluded that water intake was not associated with all-cause mortality risk in men, while compared to the first quartile, a small increased all-cause mortality risk in the highest quartile of water intake was found in women (10). Several studies also proved total fluid intake or raw water consumption did not seem to be associated with all-cause mortality or cardiovascular mortality (13, 15). Discrepancies in statistical methods, adjusted covariates, or the ethnicity of participants may lead to inconsistent conclusions. In addition, different definitions of sources of water intake would also affect the conclusion.

In this study, we found that the higher total water intake was associated with lower mortality risks due to all causes; it may be explained by the several health benefits of increased daily water intake, such as lower blood pressure, increased body temperature, diluted blood waste materials, and protected kidney function (24). Water supplementation also seemed to be an effective and safe initiative for decreasing fasting plasma glucose and reducing the risk of diabetes (25). Increased water intake could reduce the feeling of hunger, as well as stimulate sympathetic nerves and induct thermogenesis to increase lipolysis and energy depletion; thus, it is useful for weight management and obesity prevention (26–28). In addition, higher water intake may be a surrogate of healthy lifestyle habits. One study found that individuals with greater adherence to the Mediterranean diet, one of the recommended healthy dietary patterns, showed a higher water intake (29).

Our findings showed higher water intake from beverages and/or food was/were associated with lower mortality risks due to all causes and/or malignant neoplasms/cancer. Higher intakes of coffee/coffee substitutes and tea (two subgroups of beverage) were also found to be associated with lower mortality risks due to all causes and malignant neoplasms/cancer. An umbrella review included a total of 218 observational and interventional meta-analyses research and concluded coffee consumption was less likely to benefit harm than health (5). Chlorogenic acid, the most enriched antioxidant in coffee and caffeine, possesses an anticarcinogenic function by inducing enzymes involved in the activation of intracellular antioxidant defense and carcinogen detoxification. Tea consumption was also proved to be more beneficial than harmful for human health except for hot tea in a review. It partly could be explained by the antioxidative properties of polyphenols in tea (7). Moreover, these beverages could support essential water for metabolism. Food water majorly comes from fruits and vegetables; thus, higher food water intake always means higher fruit or vegetable intake. Besides, higher food water intake was associated with lower dietary energy density, which was recognized as a healthier dietary pattern (10). We surprisingly found that higher alcoholic beverage intake was associated with lower mortality risk. Alcohol has been clearly listed as a carcinogen by the WHO, and alcohol use is a major risk factor for the global disease burden (8). Compared with never/occasionally drinking, no mortality benefit was presented for low-volume alcohol consumption (30). A possible explanation is the harmful effects of alcohol may overshadow some beneficial effects, e.g., stress relief and mental pleasure. Besides, data on alcohol content in these alcoholic beverages were not available. Polyphenols, the non-alcoholic content in some alcoholic beverages, are demonstrated to enhance the antioxidant and anti-inflammatory capacity of the human body and thus, consequently have a positive impact on mortality risk reduction (31).

Most of these significant associations were seen in women but not in men, indicating that gender may modify the water intake and mortality risk associations. Differences in hormone levels may be one of the potential explanations. Angiotensin II (Ang II), a key component of the renin-angiotensin–aldosterone system to maintain proper fluid homeostasis, could increase water intake and is associated with drinking microstructure, and both ovarian and testicular hormones contribute to sex differences in the water intake response to Ang II (32). Under modulation of different hormones, adult females preferred saline intake over adult males (33), and salt intake was also associated with mortality risk. Besides, gender plays a role of modifier in the whole life span (34), and biological, behavioral, and social differences between genders are closely associated with mortality risks (35), which are supported by Kant et al. (10). More studies are needed to prove this finding.

Our study has several strengths. First, as far as we know, this is the first study to explore the associations of different kinds of water intake and water intake proportion with mortality risks due to all causes, malignant neoplasms/cancer, and heart disease, as well as the dose–response relationships among them. Second, our study had adequate representative samples and long years of follow-up. Besides, we adjusted various potential covariates to exclude as many confounders as possible, and the sensitivity analyses presented robust results. Finally, our results were stratified by gender to provide more epidemiological evidence for related fields. In addition, several limitations should be mentioned. First, although we found negative associations between water intake and mortality risks, indeed, the reverse causality bias may also exist because sick people may drink less water due to the use of medications or mobility difficulties. Healthier people may be more aware of the benefits of water intake and drink water more frequently. Furthermore, water consumption may be a surrogate of many factors, e.g., physical activity, disease status, health awareness, etc., all of which may contribute to the water intake and mortality risk association. However, similar water intake and mortality risk associations were observed by the sensitivity analyses including generally healthy participants without known diseases and those without a medical history of cancer and heart disease, indicating the stability of our findings in this study. Second, we did not control the medications used that could affect water turnover or water intake due to unavailable data. Third, self-reporting plain water intake was collected in the NHANES 1999–2004, which may not be as accurate as the data obtained by the 24-h dietary recall in the NHANES 2005–2014. Finally, our findings were obtained from the United States population. The outward implementation of our findings may be limited.

## Conclusion

We found that higher water intake was associated with lower mortality risks. Further studies are needed to prove our findings and find out the potential mechanism.

## Data Availability Statement

The datasets presented in this study can be found in online repositories. The names of the repository/repositories and accession number(s) can be found below: https://www.cdc.gov/nchs/nhanes/.

## Ethics Statement

The studies involving human participants were reviewed and approved by the Research Ethics Review Board of American National Center for Health Statistics. The patients/participants provided their written informed consent to participate in this study.

## Author Contributions

QW, JL, and HZ: conceptualization. HZ: study conduct and writing – original draft preparation. YC and MW: data collection. DD and HZ: methodology. MW, WS, and RZ: data interpretation. QW and JL: writing – review and editing and had primary responsibility for the final content. All authors read, reviewed, and approved the final manuscript.

## Conflict of Interest

The authors declare that the research was conducted in the absence of any commercial or financial relationships that could be construed as a potential conflict of interest.

## Publisher’s Note

All claims expressed in this article are solely those of the authors and do not necessarily represent those of their affiliated organizations, or those of the publisher, the editors and the reviewers. Any product that may be evaluated in this article, or claim that may be made by its manufacturer, is not guaranteed or endorsed by the publisher.

## Footnotes

1
https://wwwn.cdc.gov/Nchs/Nhanes/2013-2014/DR1TOT_H.htm

2
https://www.cdc.gov/nchs/data/series/sr_02/sr02_161.pdf

## Supplementary Material

Supplementary Figure 1 Flow chart for the selection of participants.

Supplementary Figure 2 Cluster analysis of medical history.

Supplementary Figure 3 The dose–response relationships of the amount of water intake [expressed by total water **(A)**, plain water **(B)**, beverage water **(C)**, and food water **(D)**] with the mortality risk due to all causes in men clarified by restricted cubic spline plots.

Supplementary Figure 4 The dose–response relationships of the amount of water intake [expressed by total water **(A)**, plain water **(B)**, beverage water **(C)**, and food water **(D)**] with the mortality risk due to all causes in women clarified by restricted cubic spline plots.

Supplementary Figure 5 The dose–response relationships of the amount of water intake [expressed by total water **(A)**, plain water **(B)**, beverage water **(C)**, and food water **(D)**] with the mortality risk due to malignant neoplasms/cancer in men clarified by restricted cubic spline plots.

Supplementary Figure 6 The dose–response relationships of the amount of water intake [expressed by total water **(A)**, plain water **(B)**, beverage water **(C)**, and food water **(D)**] with the mortality risk due to malignant neoplasms/cancer in women clarified by restricted cubic spline plots.

Supplementary Figure 7 The dose–response relationships of the amount of water intake [expressed by total water **(A)**, plain water **(B)**, beverage water **(C)**, and food water **(D)**] with the mortality risk due to heart diseases in men clarified by restricted cubic spline plots.

Supplementary Figure 8 The dose–response relationships of the amount of water intake [expressed by total water **(A)**, plain water **(B)**, beverage water **(C)**, and food water **(D)**] with the mortality risk due to heart diseases in women clarified by restricted cubic spline plots.

## Abbreviations

Ang II: angiotensin II
CDC: the Centers for Disease Control and Prevention
ICD: International Classification of Disease
MET: metabolic equivalent
NCHS: National Center for Health Statistics
NHANES: the National Health and Nutrition Examination Survey
PIR: family income-to-poverty ratio.
