# Supplementary figures and images for: Association Between Water Intake and Mortality Risk—Evidence From a National Prospective Study
Source: Front Nutr. 2022 Apr 12;9:822119. doi: 10.3389/fnut.2022.822119 (PMC9039539; doi:10.3389/fnut.2022.822119)

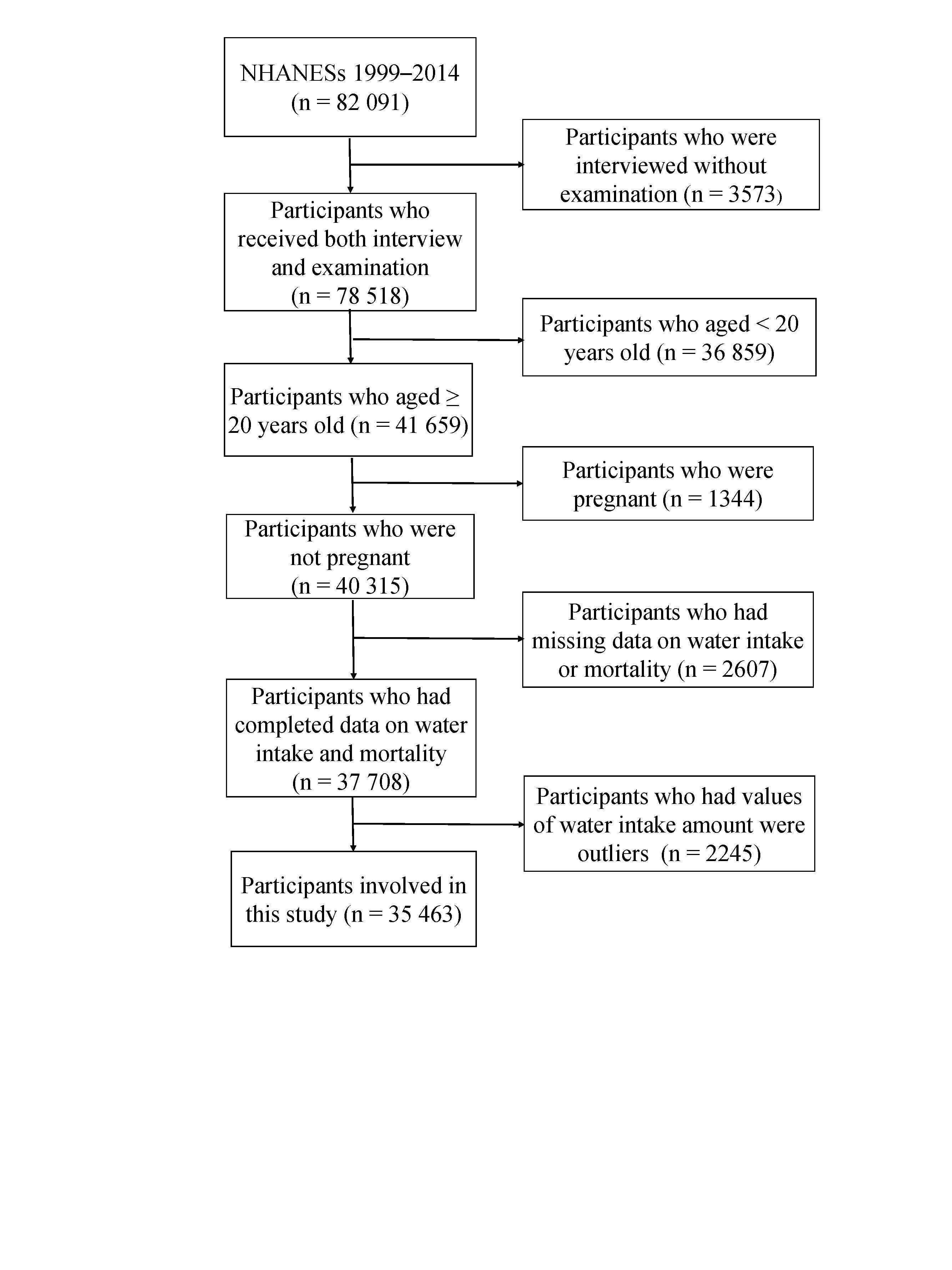

Supplement: Supplementary Figure 1 — Flow chart for the selection of participants. [file Image_1.JPEG]

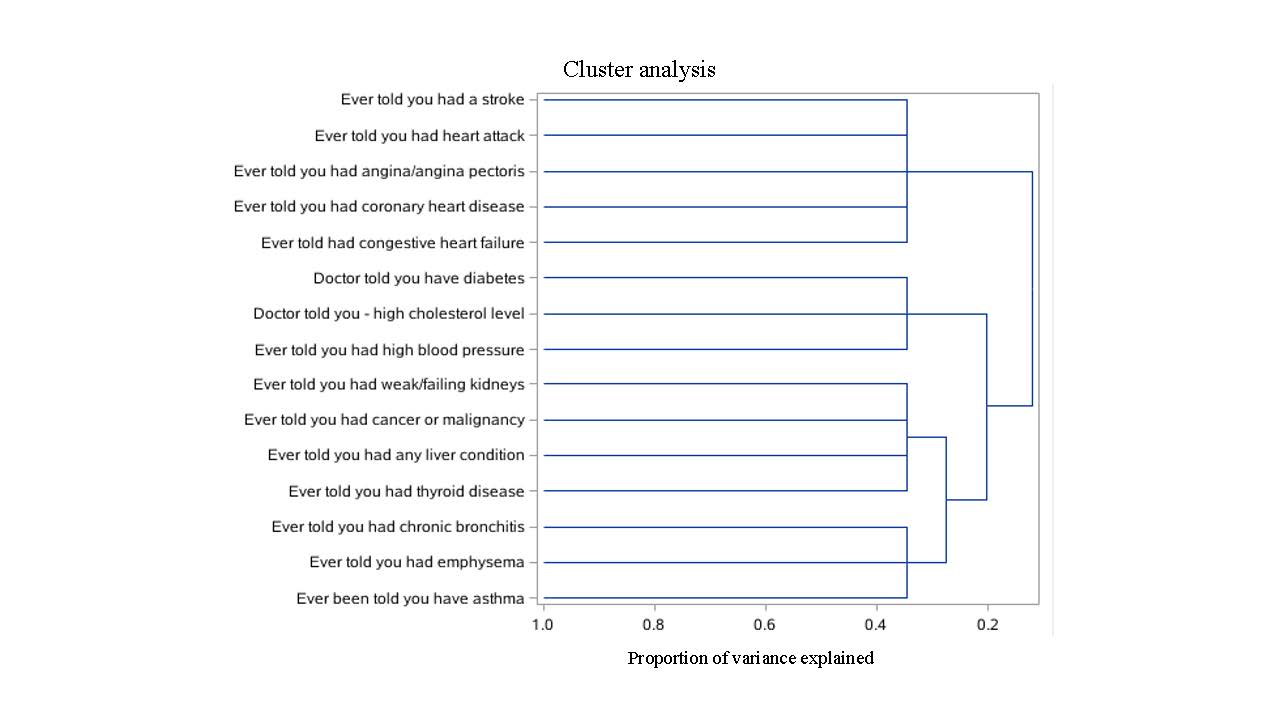

Supplement: Supplementary Figure 2 — Cluster analysis of medical history. [file Image_2.JPEG]

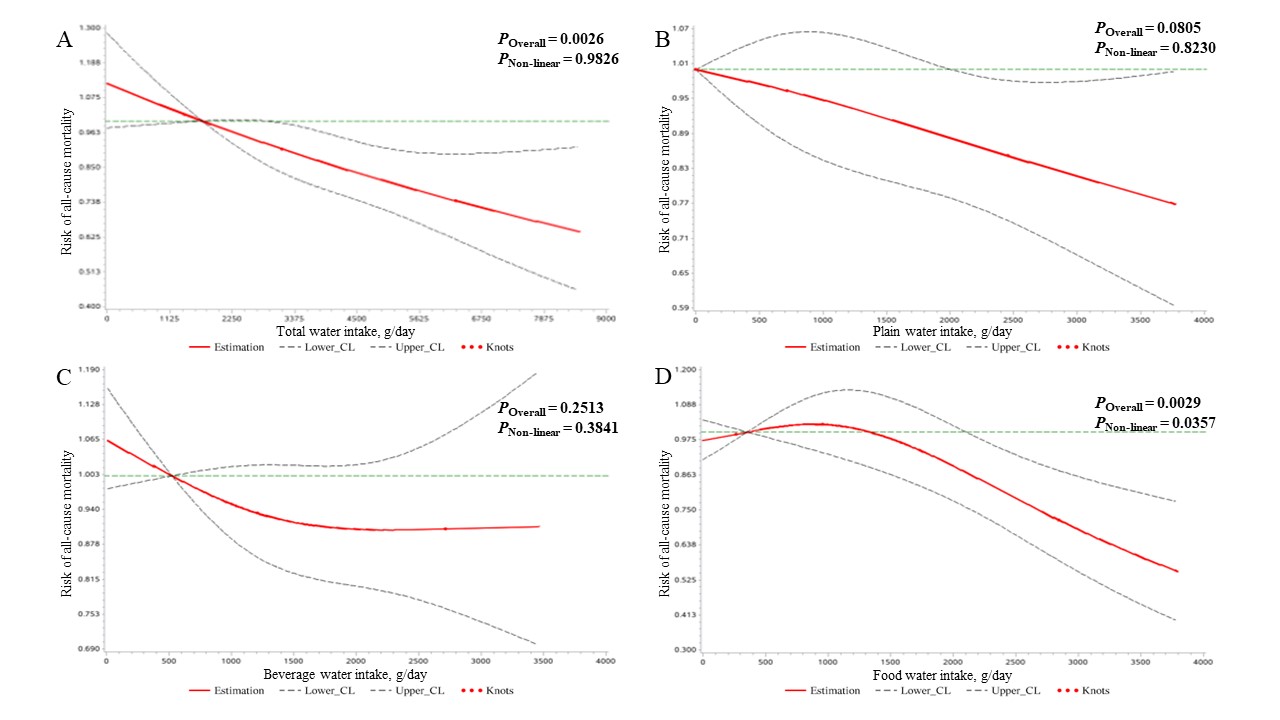

Supplement: Supplementary Figure 3 — The dose–response relationships of the amount of water intake [expressed by total water (A), plain water (B), beverage water (C), and food water (D)] with the mortality risk due to all causes in men clarified by restricted cubic spline plots. [file Image_3.jpg]

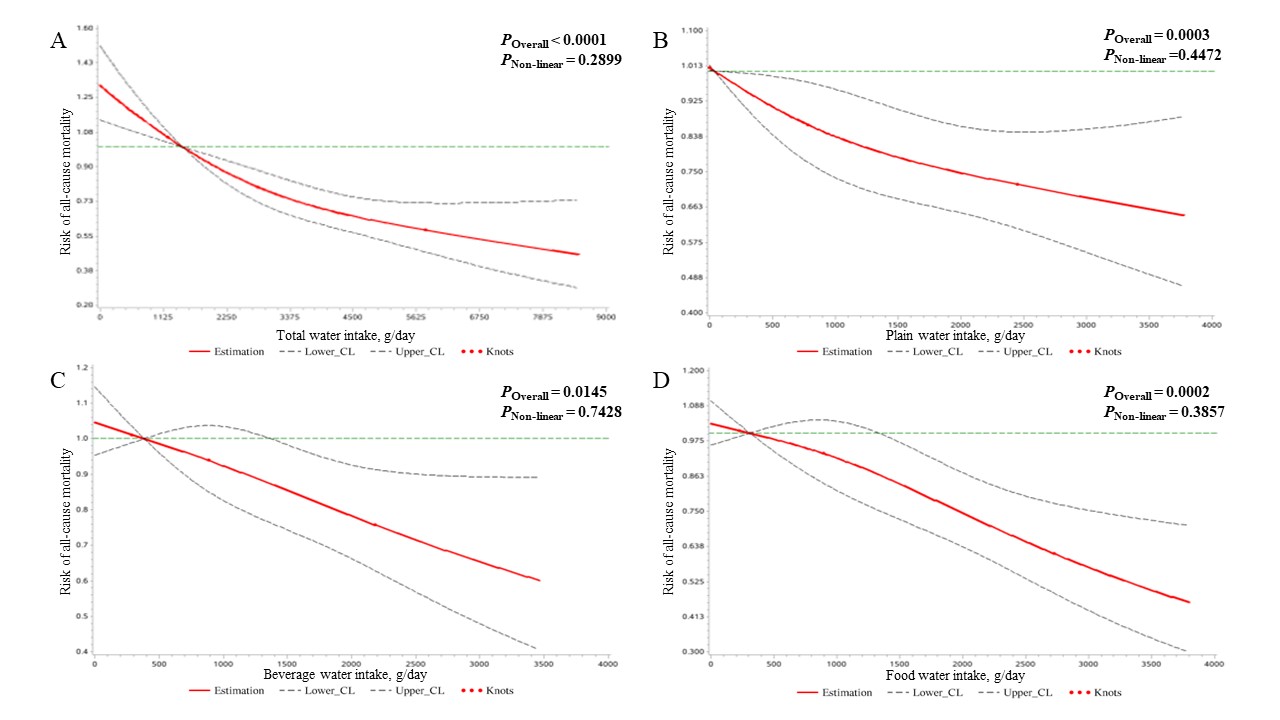

Supplement: Supplementary Figure 4 — The dose–response relationships of the amount of water intake [expressed by total water (A), plain water (B), beverage water (C), and food water (D)] with the mortality risk due to all causes in women clarified by restricted cubic spline plots. [file Image_4.jpg]

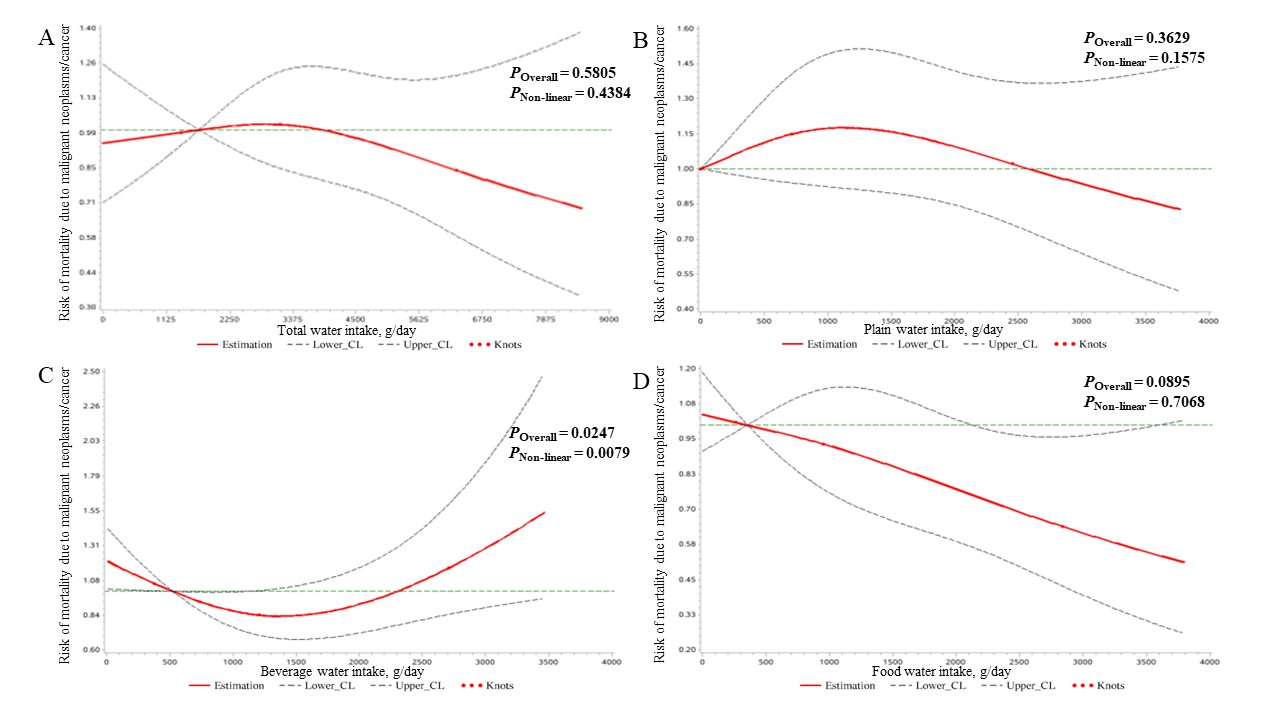

Supplement: Supplementary Figure 5 — The dose–response relationships of the amount of water intake [expressed by total water (A), plain water (B), beverage water (C), and food water (D)] with the mortality risk due to malignant neoplasms/cancer in men clarified by restricted cubic spline plots. [file Image_5.jpg]

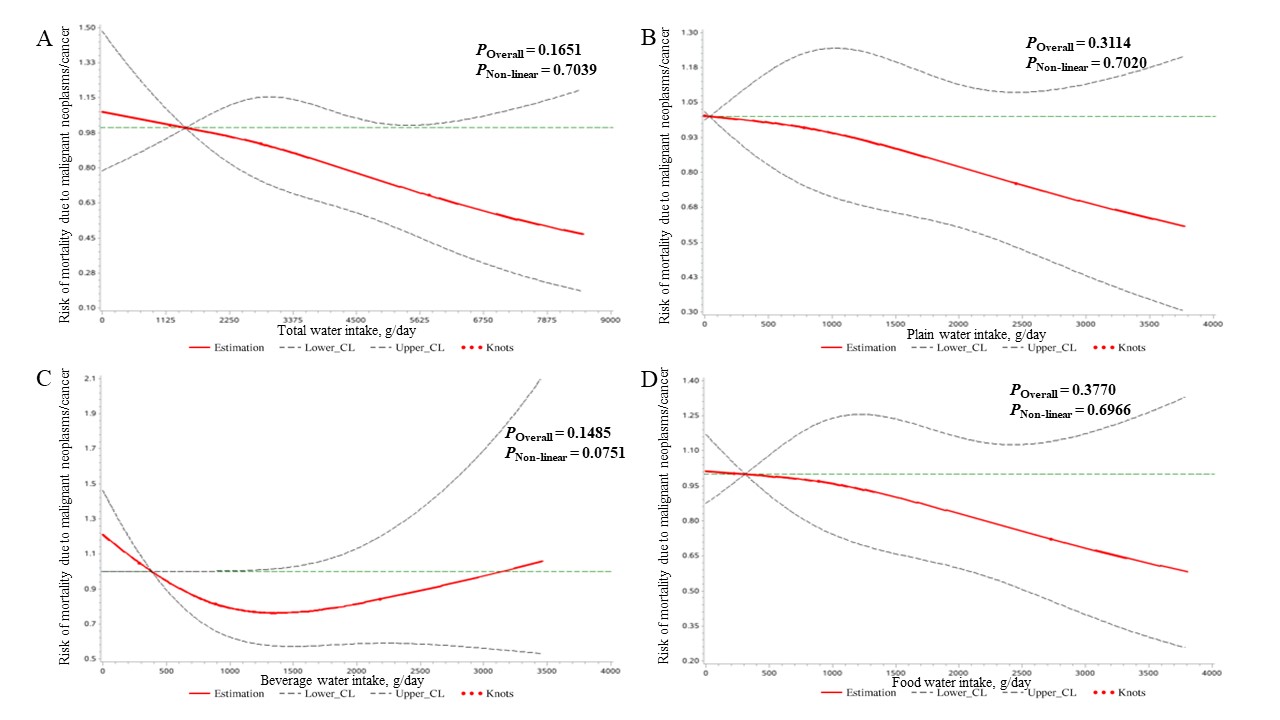

Supplement: Supplementary Figure 6 — The dose–response relationships of the amount of water intake [expressed by total water (A), plain water (B), beverage water (C), and food water (D)] with the mortality risk due to malignant neoplasms/cancer in women clarified by restricted cubic spline plots. [file Image_6.jpg]

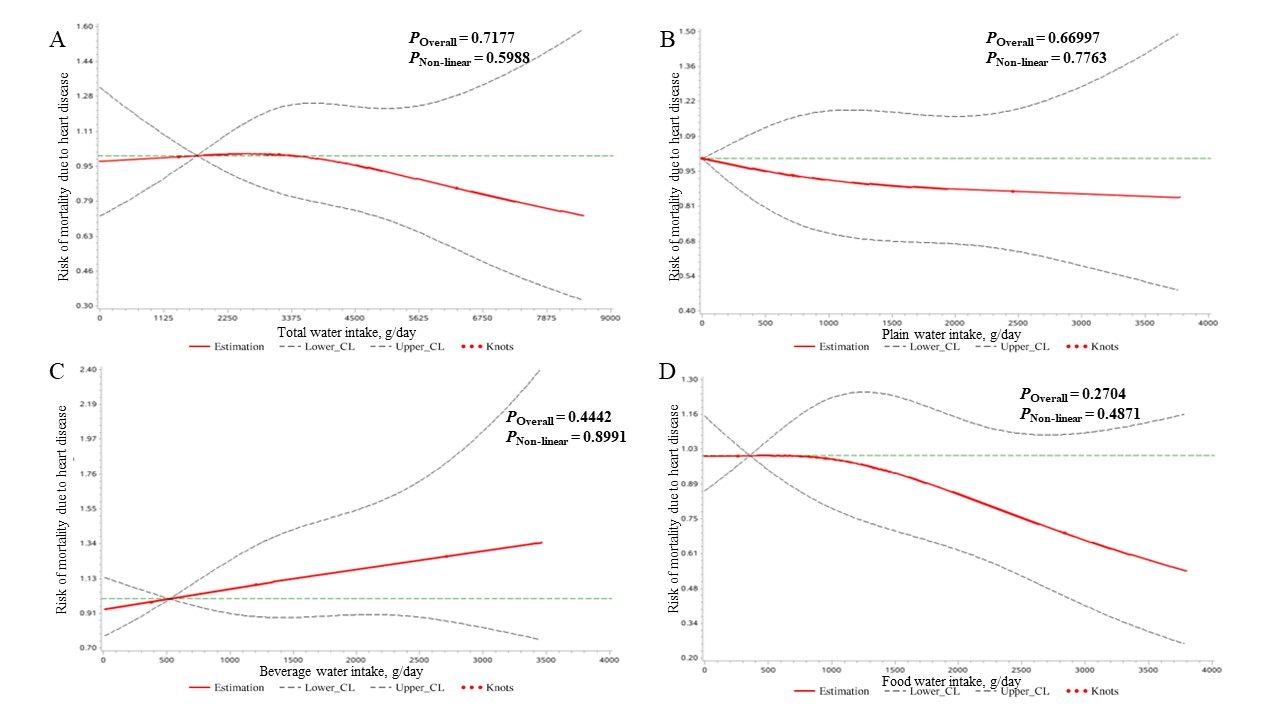

Supplement: Supplementary Figure 7 — The dose–response relationships of the amount of water intake [expressed by total water (A), plain water (B), beverage water (C), and food water (D)] with the mortality risk due to heart diseases in men clarified by restricted cubic spline plots. [file Image_7.jpg]

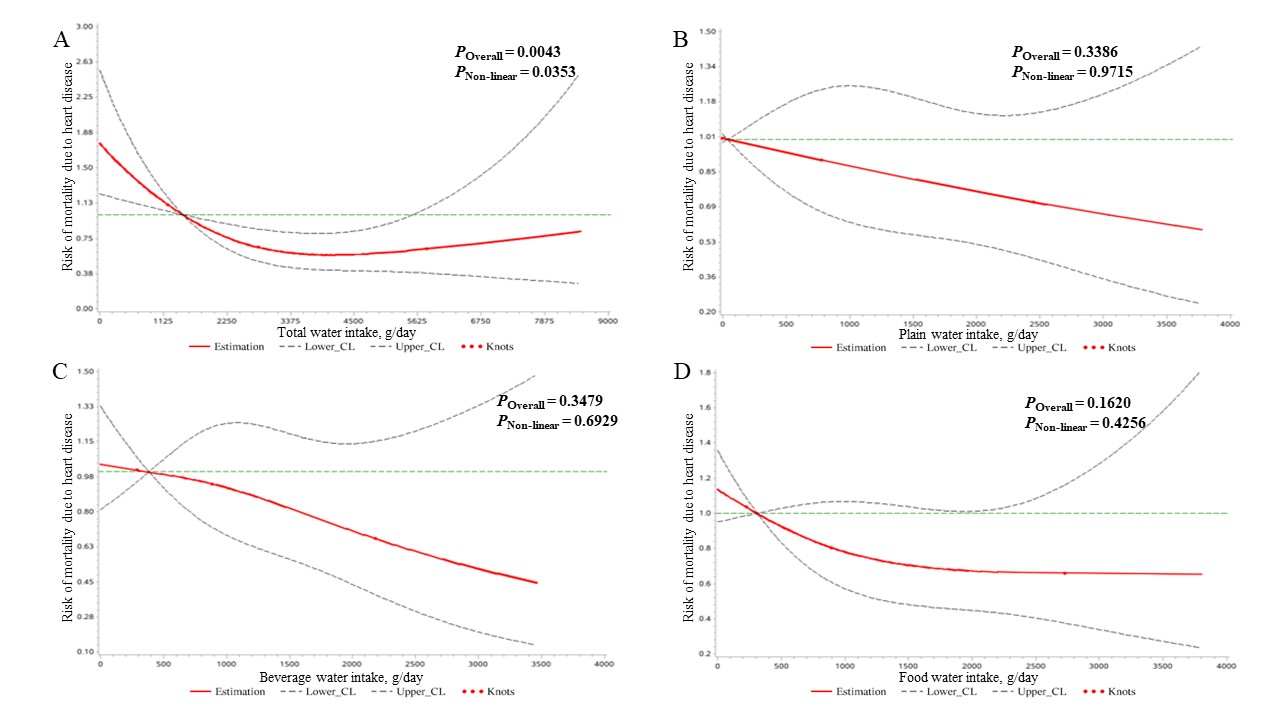

Supplement: Supplementary Figure 8 — The dose–response relationships of the amount of water intake [expressed by total water (A), plain water (B), beverage water (C), and food water (D)] with the mortality risk due to heart diseases in women clarified by restricted cubic spline plots. [file Image_8.jpg]
