# Supplementary material for: Association Between Water Intake and Mortality Risk—Evidence From a National Prospective Study
Source: Front Nutr. 2022 Apr 12;9:822119. doi: 10.3389/fnut.2022.822119 (PMC9039539; doi:10.3389/fnut.2022.822119)
Supplement: Supplementary file 9 [file Table_1.docx]

Table S1. Results of cluster analysis of self-reporting medical history.

| Four clusters | |  | R^2^ | | |
| --- | --- | --- | --- | --- | --- |
| Cluster | Variables |  | Own cluster | Next closest | 1-R^2^ ratio |
| Cluster 1 | Ever been told you had congestive heart failure |  | 0.3464 | 0.0059 | 0.6575 |
|  | Ever been told you had coronary heart disease |  | 0.4178 | 0.0027 | 0.5838 |
|  | Ever been told you had angina |  | 0.3984 | 0.0055 | 0.6049 |
|  | Ever been told you had heart attack |  | 0.3138 | 0.0108 | 0.6937 |
|  | Ever been told you had a stroke |  | 0.1278 | 0.0066 | 0.8780 |
| Cluster 2 | Ever been told you had high blood pressure |  | 0.5036 | 0.0073 | 0.5000 |
|  | Ever been told you had high cholesterol level |  | 0.3497 | 0.0039 | 0.6529 |
|  | Ever been told you had diabetes |  | 0.3901 | 0.0068 | 0.6142 |
| Cluster 3 | Ever been told you had asthma |  | 0.4356 | 0.0017 | 0.5654 |
|  | Ever been told you had emphysema |  | 0.2558 | 0.0077 | 0.7499 |
|  | Ever been told you had chronic bronchitis |  | 0.4821 | 0.0046 | 0.5203 |
| Cluster 4 | Ever been told you had thyroid disease |  | 0.2611 | 0.0049 | 0.7425 |
|  | Ever been told you had any liver condition |  | 0.3579 | 0.0039 | 0.6446 |
|  | Ever been told you had cancer or malignancy |  | 0.2628 | 0.0073 | 0.7426 |
|  | Ever been told you had weak/failing kidneys |  | 0.2776 | 0.0046 | 0.7257 |

Table S2. Associations of total water, plain water, beverage water and food water intake with all-cause mortality risk in the NHANESs 1999–2014 men.

| Water intake | 1^st^ quartile | 2^nd^ quartile | 3^rd^ quartile | 4^th^ quartile |
| --- | --- | --- | --- | --- |
| **Model 1** |  |  |  |  |
| Total water | 1.00 | 0.91 (0.78, 1.06) | **0.79 (0.69, 0.91)** | **0.77 (0.63, 0.94)** |
| Plain water | 1.00 | 1.06 (0.87, 1.28) | 0.88 (0.73, 1.07) | 0.94 (0.77, 1.14) |
| Beverage water | 1.00 | **0.84 (0.73, 0.96)** | **0.81 (0.69, 0.96)** | **0.76 (0.65, 0.90)** |
| Food water | 1.00 | 0.90 (0.77, 1.05) | **0.80 (0.67, 0.95)** | **0.77 (0.61, 0.97)** |
| **Model 2** |  |  |  |  |
| Total water | 1.00 | 1.01 (0.86, 1.18) | 0.93 (0.81, 1.07) | 0.94 (0.75, 1.17) |
| Plain water | 1.00 | 1.09 (0.91, 1.31) | 0.95 (0.79, 1.13) | 1.02 (0.84, 1.24) |
| Beverage water | 1.00 | 0.88 (0.76, 1.02) | 0.89 (0.75, 1.06) | 0.87 (0.73, 1.04) |
| Food water | 1.00 | 1.01 (0.86, 1.18) | 0.93 (0.77, 1.13) | 0.89 (0.69, 1.15) |
| **Model 3** |  |  |  |  |
| Total water | 1.00 | 0.99 (0.85, 1.15) | 0.92 (0.80, 1.06) | 0.90 (0.72, 1.13) |
| Plain water | 1.00 | 1.05 (0.87, 1.26) | 0.91 (0.76, 1.10) | 1.00 (0.82, 1.22) |
| Beverage water | 1.00 | 0.89 (0.76, 1.05) | 0.92 (0.76, 1.10) | 0.89 (0.74, 1.08) |
| Food water | 1.00 | 0.99 (0.85, 1.15) | 0.92 (0.76, 1.12) | 0.88 (0.68, 1.13) |
| **Model 4** |  |  |  |  |
| Total water | - | - | - | - |
| Plain water | 1.00 | 1.08 (0.89, 1.30) | 0.93 (0.78, 1.12) | 1.02 (0.84, 1.25) |
| Beverage water | 1.00 | 0.88 (0.75, 1.04) | 0.90 (0.75, 1.08) | 0.87 (0.72, 1.06) |
| Food water | 1.00 | 0.97 (0.83, 1.13) | 0.90 (0.74, 1.10) | 0.87 (0.66, 1.14) |

NHANES, National Health and Nutrition Examination Survey.

All estimates were calculated by multivariable Cox proportional hazards regression models, and results were expressed as hazard ratio (95% confidence interval). Total water intake is the sum of water intake from plain water, beverage and food. Four kinds of water intake were categorized into four groups according to respective quartiles. Covariates adjusted in model 1 included age, race, education, marital status, ratio of family income to poverty, body mass index. Covariates adjusted in model 2 included leisure time physical activity, dietary total energy, dietary protein, dietary carbohydrate, dietary total fat, dietary fiber, drinking status and smoking status in addition to those in model 1. Covariates adjusted in model 3 included cluster 1–4 of medical history in addition to those in model 2. Covariates adjusted in model 4 included the other kinds of water source in addition to those in model 3. That is, for plain water, we included beverage water and food water as added covariates; for beverage water, we included plain water and food water as added covariates; and for food water, we included plain water and beverage water as added covariates.

The range of quartiles for total water intake were < 2310 (Q1), 2310–3149 (Q2), 3150–4241 (Q3) and 4242–8516 g/day (Q4). The range of quartiles for plain water intake were < 281 (Q1), 281–718 (Q2), 719–1342 (Q3) and 1343–3776 g/day (Q4). The range of quartiles for beverage water intake were < 806 (Q1), 806–1209 (Q2), 1210–1746 (Q3) and 1747–3464 g/day (Q4). The range of quartiles for food water intake were < 566 (Q1), 567–954 (Q2), 955–1637 (Q3) and 1638–3795 g/day (Q4).

Boldness indicates a statistical significance.

Table S3. Associations of total water, plain water, beverage water and food water intake with all-cause mortality risk in the NHANESs 1999–2014 women.

| Water intake | 1^st^ quartile | 2^nd^ quartile | 3^rd^ quartile | 4^th^ quartile |
| --- | --- | --- | --- | --- |
| **Model 1** |  |  |  |  |
| Total water | 1.00 | **0.74 (0.62, 0.89)** | **0.59 (0.49, 0.71)** | **0.57 (0.47, 0.70)** |
| Plain water | 1.00 | **0.84 (0.73, 0.97)** | **0.70 (0.60, 0.83)** | **0.76 (0.64, 0.92)** |
| Beverage water | 1.00 | 0.91 (0.78, 1.07) | 0.87 (0.71, 1.07) | **0.70 (0.58, 0.84)** |
| Food water | 1.00 | 0.98 (0.84, 1.16) | **0.79 (0.67, 0.93)** | **0.62 (0.49, 0.78)** |
| **Model 2** |  |  |  |  |
| Total water | 1.00 | **0.80 (0.67, 0.95)** | **0.67 (0.55, 0.81)** | **0.66 (0.54, 0.82)** |
| Plain water | 1.00 | 0.94 (0.82, 1.08) | **0.77 (0.65, 0.92)** | 0.84 (0.70, 1.02) |
| Beverage water | 1.00 | 0.93 (0.80, 1.08) | 0.89 (0.72, 1.11) | **0.71 (0.58, 0.88)** |
| Food water | 1.00 | 1.17 (0.99, 1.39) | 0.97 (0.81, 1.17) | **0.76 (0.60, 0.97)** |
| **Model 3** |  |  |  |  |
| Total water | 1.00 | **0.83 (0.70, 0.99)** | **0.65 (0.54, 0.80)** | **0.65 (0.53, 0.80)** |
| Plain water | 1.00 | 0.90 (0.78, 1.04) | **0.75 (0.63, 0.89)** | **0.78 (0.64, 0.93)** |
| Beverage water | 1.00 | 0.89 (0.77, 1.03) | 0.89 (0.71, 1.10) | **0.71 (0.57, 0.89)** |
| Food water | 1.00 | 1.14 (0.96, 1.36) | 0.97 (0.81, 1.16) | **0.77 (0.60, 0.98)** |
| **Model 4** |  |  |  |  |
| Total water | - | - | - | - |
| Plain water | 1.00 | 0.90 (0.77, 1.04) | **0.78 (0.65, 0.93)** | 0.83 (0.68, 1.01) |
| Beverage water | 1.00 | **0.86 (0.74, 1.00)** | 0.85 (0.69, 1.06) | **0.68 (0.54, 0.85)** |
| Food water | 1.00 | 1.12 (0.93, 1.33) | 0.96 (0.79, 1.16) | 0.80 (0.62, 1.01) |

NHANES, National Health and Nutrition Examination Survey.

All estimates were calculated by multivariable Cox proportional hazards regression models, and results were expressed as hazard ratio (95% confidence interval). Total water intake is the sum of water intake from plain water, beverage and food. Four kinds of water intake were categorized into four groups according to respective quartiles. Covariates adjusted in model 1 included age, race, education, marital status, ratio of family income to poverty, body mass index. Covariates adjusted in model 2 included leisure time physical activity, dietary total energy, dietary protein, dietary carbohydrate, dietary total fat, dietary fiber, drinking status and smoking status in addition to those in model 1. Covariates adjusted in model 3 included cluster 1–4 of medical history in addition to those in model 2. Covariates adjusted in model 4 included the other kinds of water source in addition to those in model 3. That is, for plain water, we included beverage water and food water as added covariates; for beverage water, we included plain water and food water as added covariates; and for food water, we included plain water and beverage water as added covariates.

The range of quartiles for total water intake were < 2019 (Q1), 2019–2800 (Q2), 2801–3841 (Q3) and 3842–8511 g/day (Q4). The range of quartiles for plain water intake were < 333 (Q1), 333–781 (Q2), 782–1406 (Q3) and 1407–3776 g/day (Q4). The range of quartiles for beverage water intake were < 595 (Q1), 595–885 (Q2), 886–1293 (Q3) and 1294–3464 g/day (Q4). The range of quartiles for food water intake were < 499 (Q1), 499–891 (Q2), 892–1592 (Q3) and 1593–3802 g/day (Q4).

Boldness indicates a statistical significance.

Table S4. Associations of total water, plain water, beverage water and food water intake with malignant neoplasms/cancer mortality risk in the NHANESs 1999–2014 men.

| Water intake | 1^st^ quartile | 2^nd^ quartile | 3^rd^ quartile | 4^th^ quartile |
| --- | --- | --- | --- | --- |
| **Model 1** |  |  |  |  |
| Total water | 1.00 | 1.19 (0.91, 1.57) | 1.09 (0.81, 1.46) | 1.10 (0.78, 1.55) |
| Plain water | 1.00 | 1.02 (0.75, 1.40) | 0.92 (0.67, 1.26) | 0.92 (0.66, 1.28) |
| Beverage water | 1.00 | 0.89 (0.65, 1.21) | 0.95 (0.66, 1.38) | 1.33 (0.94, 1.89) |
| Food water | 1.00 | 1.02 (0.75, 1.38) | 0.78 (0.55, 1.10) | **0.57 (0.37, 0.86)** |
| **Model 2** |  |  |  |  |
| Total water | 1.00 | 1.33 (0.99, 1.79) | 1.31 (0.95, 1.81) | 1.37 (0.94, 1.98) |
| Plain water | 1.00 | 1.06 (0.78, 1.45) | 1.01 (0.73, 1.39) | 1.04 (0.74, 1.47) |
| Beverage water | 1.00 | 0.89 (0.65, 1.22) | 0.99 (0.69, 1.42) | 1.37 (0.93, 2.02) |
| Food water | 1.00 | 1.20 (0.87, 1.65) | 0.99 (0.69, 1.42) | 0.74 (0.49, 1.12) |
| **Model 3** |  |  |  |  |
| Total water | 1.00 | 1.32 (0.98, 1.77) | 1.33 (0.96, 1.84) | 1.36 (0.95, 1.96) |
| Plain water | 1.00 | 1.04 (0.76, 1.43) | 0.98 (0.71, 1.36) | 1.03 (0.74, 1.45) |
| Beverage water | 1.00 | 0.90 (0.65, 1.25) | 1.01 (0.70, 1.47) | 1.41 (0.95, 2.10) |
| Food water | 1.00 | 1.20 (0.86, 1.65) | 1.00 (0.69, 1.44) | 0.75 (0.49, 1.14) |
| **Model 4** |  |  |  |  |
| Total water | - | - | - | - |
| Plain water | 1.00 | 1.10 (0.79, 1.54) | 1.11 (0.80, 1.55) | 1.24 (0.84, 1.83) |
| Beverage water | 1.00 | 0.90 (0.65, 1.24) | 1.00 (0.69, 1.45) | 1.39 (0.93, 2.07) |
| Food water | 1.00 | 1.20 (0.87, 1.65) | 1.01 (0.69, 1.47) | 0.72 (0.46, 1.14) |

NHANES, National Health and Nutrition Examination Survey.

All estimates were calculated by multivariable Cox proportional hazards regression models, and results were expressed as hazard ratio (95% confidence interval). Total water intake is the sum of water intake from plain water, beverage and food. Four kinds of water intake were categorized into four groups according to respective quartiles. Covariates adjusted in model 1 included age, race, education, marital status, ratio of family income to poverty, body mass index. Covariates adjusted in model 2 included leisure time physical activity, dietary total energy, dietary protein, dietary carbohydrate, dietary total fat, dietary fiber, drinking status and smoking status in addition to those in model 1. Covariates adjusted in model 3 included cluster 1–4 of medical history in addition to those in model 2. Covariates adjusted in model 4 included the other kinds of water source in addition to those in model 3. That is, for plain water, we included beverage water and food water as added covariates; for beverage water, we included plain water and food water as added covariates; and for food water, we included plain water and beverage water as added covariates.

The range of quartiles for total water intake were < 2310 (Q1), 2310–3149 (Q2), 3150–4241 (Q3) and 4242–8516 g/day (Q4). The range of quartiles for plain water intake were < 281 (Q1), 281–718 (Q2), 719–1342 (Q3) and 1343–3776 g/day (Q4). The range of quartiles for beverage water intake were < 806 (Q1), 806–1209 (Q2), 1210–1746 (Q3) and 1747–3464 g/day (Q4). The range of quartiles for food water intake were < 566 (Q1), 567–954 (Q2), 955–1637 (Q3) and 1638–3795 g/day (Q4).

Boldness indicates a statistical significance.

Table S5. Associations of total water, plain water, beverage water and food water intake with malignant neoplasms/cancer mortality risk in the NHANESs 1999–2014 women.

| Water intake | 1^st^ quartile | 2^nd^ quartile | 3^rd^ quartile | 4^th^ quartile |
| --- | --- | --- | --- | --- |
| **Model 1** |  |  |  |  |
| Total water | 1.00 | 0.83 (0.56, 1.21) | 0.67 (0.45, 1.01) | **0.53 (0.34, 0.83)** |
| Plain water | 1.00 | 0.78 (0.56, 1.10) | **0.69 (0.49, 0.98)** | 0.75 (0.48, 1.15) |
| Beverage water | 1.00 | 0.95 (0.66, 1.35) | 0.71 (0.45, 1.13) | 0.72 (0.48, 1.06) |
| Food water | 1.00 | 0.88 (0.65, 1.19) | 0.83 (0.57, 1.21) | **0.61 (0.39, 0.96)** |
| **Model 2** |  |  |  |  |
| Total water | 1.00 | 0.84 (0.57, 1.26) | 0.71 (0.47, 1.09) | **0.56 (0.35, 0.89)** |
| Plain water | 1.00 | 0.88 (0.63, 1.22) | 0.75 (0.54, 1.06) | 0.81 (0.52, 1.27) |
| Beverage water | 1.00 | 0.94 (0.65, 1.35) | 0.66 (0.40, 1.10) | **0.62 (0.4, 0.97)** |
| Food water | 1.00 | 1.02 (0.73, 1.44) | 0.99 (0.65, 1.50) | 0.74 (0.45, 1.20) |
| **Model 3** |  |  |  |  |
| Total water | 1.00 | 0.84 (0.56, 1.26) | 0.69 (0.46, 1.06) | **0.54 (0.34, 0.86)** |
| Plain water | 1.00 | 0.85 (0.60, 1.20) | 0.73 (0.52, 1.04) | 0.78 (0.49, 1.24) |
| Beverage water | 1.00 | 0.91 (0.64, 1.31) | 0.65 (0.39, 1.08) | **0.61 (0.39, 0.97)** |
| Food water | 1.00 | 1.02 (0.72, 1.43) | 0.98 (0.65, 1.48) | 0.73 (0.44, 1.20) |
| **Model 4** |  |  |  |  |
| Total water | - | - | - | - |
| Plain water | 1.00 | 0.82 (0.56, 1.19) | 0.74 (0.52, 1.05) | 0.81 (0.49, 1.35) |
| Beverage water | 1.00 | 0.89 (0.62, 1.28) | 0.62 (0.37, 1.04) | **0.58 (0.36, 0.92)** |
| Food water | 1.00 | 0.99 (0.69, 1.40) | 0.99 (0.63, 1.56) | 0.73 (0.44, 1.23) |

NHANES, National Health and Nutrition Examination Survey.

All estimates were calculated by multivariable Cox proportional hazards regression models, and results were expressed as hazard ratio (95% confidence interval). Total water intake is the sum of water intake from plain water, beverage and food. Four kinds of water intake were categorized into four groups according to respective quartiles. Covariates adjusted in model 1 included age, race, education, marital status, ratio of family income to poverty, body mass index. Covariates adjusted in model 2 included leisure time physical activity, dietary total energy, dietary protein, dietary carbohydrate, dietary total fat, dietary fiber, drinking status and smoking status in addition to those in model 1. Covariates adjusted in model 3 included cluster 1–4 of medical history in addition to those in model 2. Covariates adjusted in model 4 included the other kinds of water source in addition to those in model 3. That is, for plain water, we included beverage water and food water as added covariates; for beverage water, we included plain water and food water as added covariates; and for food water, we included plain water and beverage water as added covariates.

The range of quartiles for total water intake were < 2019 (Q1), 2019–2800 (Q2), 2801–3841 (Q3) and 3842–8511 g/day (Q4). The range of quartiles for plain water intake were < 333 (Q1), 333–781 (Q2), 782–1406 (Q3) and 1407–3776 g/day (Q4). The range of quartiles for beverage water intake were < 595 (Q1), 595–885 (Q2), 886–1293 (Q3) and 1294–3464 g/day (Q4). The range of quartiles for food water intake were < 499 (Q1), 499–891 (Q2), 892–1592 (Q3) and 1593–3802 g/day (Q4).

Boldness indicates a statistical significance.

Table S6. Associations of total water, plain water, beverage water and food water intake with heart disease mortality risk in the NHANESs 1999–2014 men.

| Water intake | 1^st^ quartile | 2^nd^ quartile | 3^rd^ quartile | 4^th^ quartile |
| --- | --- | --- | --- | --- |
| **Model 1** |  |  |  |  |
| Total water | 1.00 | 0.82 (0.61, 1.11) | 0.83 (0.61, 1.13) | **0.54 (0.35, 0.85)** |
| Plain water | 1.00 | 1.08 (0.78, 1.51) | 0.82 (0.54, 1.25) | 1.03 (0.72, 1.45) |
| Beverage water | 1.00 | 0.85 (0.56, 1.27) | 0.73 (0.48, 1.12) | **0.62 (0.40, 0.96)** |
| Food water | 1.00 | 0.76 (0.57, 1.01) | 0.79 (0.57, 1.10) | 0.87 (0.53, 1.42) |
| **Model 2** |  |  |  |  |
| Total water | 1.00 | 0.99 (0.74, 1.32) | 1.07 (0.76, 1.52) | 0.75 (0.47, 1.20) |
| Plain water | 1.00 | 1.11 (0.80, 1.53) | 0.87 (0.57, 1.34) | 1.11 (0.77, 1.61) |
| Beverage water | 1.00 | 0.99 (0.66, 1.49) | 0.93 (0.63, 1.39) | 0.91 (0.57, 1.44) |
| Food water | 1.00 | 0.88 (0.65, 1.21) | 0.95 (0.66, 1.37) | 1.00 (0.63, 1.59) |
| **Model 3** |  |  |  |  |
| Total water | 1.00 | 0.96 (0.72, 1.27) | 1.04 (0.74, 1.46) | 0.70 (0.43, 1.12) |
| Plain water | 1.00 | 1.02 (0.74, 1.41) | 0.82 (0.54, 1.24) | 1.06 (0.73, 1.54) |
| Beverage water | 1.00 | 1.03 (0.69, 1.55) | 1.00 (0.67, 1.50) | 0.97 (0.61, 1.54) |
| Food water | 1.00 | 0.87 (0.63, 1.18) | 0.91 (0.64, 1.30) | 0.95 (0.61, 1.50) |
| **Model 4** |  |  |  |  |
| Total water | - | - | - | - |
| Plain water | 1.00 | 1.03 (0.73, 1.44) | 0.81 (0.52, 1.25) | 1.05 (0.70, 1.58) |
| Beverage water | 1.00 | 1.03 (0.68, 1.56) | 1.00 (0.66, 1.50) | 0.96 (0.60, 1.53) |
| Food water | 1.00 | 0.85 (0.63, 1.16) | 0.92 (0.63, 1.34) | 0.96 (0.59, 1.55) |

NHANES, National Health and Nutrition Examination Survey.

All estimates were calculated by multivariable Cox proportional hazards regression models, and results were expressed as hazard ratio (95% confidence interval). Total water intake is the sum of water intake from plain water, beverage and food. Four kinds of water intake were categorized into four groups according to respective quartiles. Covariates adjusted in model 1 included age, race, education, marital status, ratio of family income to poverty, body mass index. Covariates adjusted in model 2 included leisure time physical activity, dietary total energy, dietary protein, dietary carbohydrate, dietary total fat, dietary fiber, drinking status and smoking status in addition to those in model 1. Covariates adjusted in model 3 included cluster 1–4 of medical history in addition to those in model 2. Covariates adjusted in model 4 included the other kinds of water source in addition to those in model 3. That is, for plain water, we included beverage water and food water as added covariates; for beverage water, we included plain water and food water as added covariates; and for food water, we included plain water and beverage water as added covariates.

The range of quartiles for total water intake were < 2310 (Q1), 2310–3149 (Q2), 3150–4241 (Q3) and 4242–8516 g/day (Q4). The range of quartiles for plain water intake were < 281 (Q1), 281–718 (Q2), 719–1342 (Q3) and 1343–3776 g/day (Q4). The range of quartiles for beverage water intake were < 806 (Q1), 806–1209 (Q2), 1210–1746 (Q3) and 1747–3464 g/day (Q4). The range of quartiles for food water intake were < 566 (Q1), 567–954 (Q2), 955–1637 (Q3) and 1638–3795 g/day (Q4).

Boldness indicates a statistical significance.

Table S7. Associations of total water, plain water, beverage water and food water intake with heart disease mortality risk in the NHANESs 1999–2014 women.

| Water intake | 1^st^ quartile | 2^nd^ quartile | 3^rd^ quartile | 4^th^ quartile |
| --- | --- | --- | --- | --- |
| **Model 1** |  |  |  |  |
| Total water | 1.00 | 0.87 (0.56, 1.36) | 0.63 (0.38, 1.02) | **0.47 (0.27, 0.82)** |
| Plain water | 1.00 | 0.88 (0.58, 1.35) | **0.63 (0.40, 0.98)** | 0.72 (0.45, 1.16) |
| Beverage water | 1.00 | 0.79 (0.49, 1.27) | 0.75 (0.45, 1.25) | 0.64 (0.40, 1.00) |
| Food water | 1.00 | 0.92 (0.61, 1.39) | 0.71 (0.42, 1.20) | 0.57 (0.32, 1.03) |
| **Model 2** |  |  |  |  |
| Total water | 1.00 | 0.95 (0.61, 1.48) | 0.72 (0.43, 1.19) | **0.59 (0.34, 1.00)** |
| Plain water | 1.00 | 0.98 (0.64, 1.49) | 0.67 (0.41, 1.09) | 0.78 (0.47, 1.30) |
| Beverage water | 1.00 | 0.85 (0.54, 1.32) | 0.82 (0.49, 1.38) | 0.73 (0.43, 1.22) |
| Food water | 1.00 | 1.05 (0.65, 1.69) | 0.86 (0.50, 1.48) | 0.68 (0.37, 1.26) |
| **Model 3** |  |  |  |  |
| Total water | 1.00 | 1.03 (0.67, 1.60) | 0.74 (0.45, 1.23) | 0.61 (0.36, 1.03) |
| Plain water | 1.00 | 0.94 (0.61, 1.45) | 0.69 (0.43, 1.10) | 0.74 (0.45, 1.22) |
| Beverage water | 1.00 | 0.80 (0.52, 1.24) | 0.83 (0.49, 1.41) | 0.77 (0.44, 1.33) |
| Food water | 1.00 | 0.95 (0.59, 1.54) | 0.83 (0.48, 1.44) | 0.67 (0.37, 1.21) |
| **Model 4** |  |  |  |  |
| Total water | - | - | - | - |
| Plain water | 1.00 | 0.98 (0.62, 1.54) | 0.72 (0.45, 1.15) | 0.80 (0.46, 1.38) |
| Beverage water | 1.00 | 0.76 (0.49, 1.20) | 0.79 (0.46, 1.36) | 0.71 (0.40, 1.24) |
| Food water | 1.00 | 0.91 (0.56, 1.48) | 0.80 (0.44, 1.43) | 0.72 (0.39, 1.32) |

NHANES, National Health and Nutrition Examination Survey.

All estimates were calculated by multivariable Cox proportional hazards regression models, and results were expressed as hazard ratio (95% confidence interval). Total water intake is the sum of water intake from plain water, beverage and food. Four kinds of water intake were categorized into four groups according to respective quartiles. Covariates adjusted in model 1 included age, race, education, marital status, ratio of family income to poverty, body mass index. Covariates adjusted in model 2 included leisure time physical activity, dietary total energy, dietary protein, dietary carbohydrate, dietary total fat, dietary fiber, drinking status and smoking status in addition to those in model 1. Covariates adjusted in model 3 included cluster 1–4 of medical history in addition to those in model 2. Covariates adjusted in model 4 included the other kinds of water source in addition to those in model 3. That is, for plain water, we included beverage water and food water as added covariates; for beverage water, we included plain water and food water as added covariates; and for food water, we included plain water and beverage water as added covariates.

The range of quartiles for total water intake were < 2019 (Q1), 2019–2800 (Q2), 2801–3841 (Q3) and 3842–8511 g/day (Q4). The range of quartiles for plain water intake were < 333 (Q1), 333–781 (Q2), 782–1406 (Q3) and 1407–3776 g/day (Q4). The range of quartiles for beverage water intake were < 595 (Q1), 595–885 (Q2), 886–1293 (Q3) and 1294–3464 g/day (Q4). The range of quartiles for food water intake were < 499 (Q1), 499–891 (Q2), 892–1592 (Q3) and 1593–3802 g/day (Q4).

Boldness indicates a statistical significance.

Table S8. Associations of water intake proportions with mortality risks due to all causes, malignant neoplasms/cancer and heart diseases in all NHANES participants, men and women.

| Water intake proportion | | Total | Men | Women |
| --- | --- | --- | --- | --- |
|  |  | *All cause mortality* | | |
| **Model 1** | Plain water | 1.000  (0.998, 1.002) | 1.001  (0.997, 1.004) | 1.000  (0.996, 1.003) |
|  | Beverage water | 1.001  (0.999, 1.003) | 1.000  (0.997, 1.004) | 1.002  (0.998, 1.005) |
|  | Food water | 0.998  (0.995, 1.001) | 0.997  (0.993, 1.002) | 0.998  (0.994, 1.002) |
| **Model 2** | Plain water | 1.000  (0.998, 1.003) | 1.000  (0.997, 1.004) | 1.000  (0.996, 1.003) |
|  | Beverage water | 1.000  (0.997, 1.002) | 1.000  (0.996, 1.003) | 1.000  (0.996, 1.003) |
|  | Food water | 1.000  (0.997, 1.004) | 0.999  (0.994, 1.004) | 1.002  (0.998, 1.007) |
| **Model 3** | Plain water | 0.999  (0.997, 1.001) | 1.000  (0.996, 1.003) | 0.998  (0.994, 1.002) |
|  | Beverage water | 1.000  (0.998, 1.003) | 1.000  (0.997, 1.004) | 1.001  (0.997, 1.004) |
|  | Food water | 1.001  (0.998, 1.004) | 0.999  (0.994, 1.004) | 1.003  (0.998, 1.008) |
|  |  | *Malignant neoplasms/cancer mortality risk* | | |
| **Model 1** | Plain water | 0.999  (0.994, 1.004) | 1.000  (0.994, 1.006) | 0.999  (0.990, 1.007) |
|  | Beverage water | 1.005  (1.001, 1.010) | **1.007**  **(1.000, 1.014)** | 1.003  (0.996, 1.010) |
|  | Food water | **0.989**  **(0.981, 0.997)** | **0.984**  **(0.973, 0.994)** | 0.995  (0.985, 1.006) |
| **Model 2** | Plain water | 1.001  (0.996, 1.006) | 1.001  (0.996, 1.008) | 0.999  (0.991, 1.008) |
|  | Beverage water | 1.002  (0.997, 1.006) | 1.003  (0.996, 1.010) | 1.000  (0.992, 1.007) |
|  | Food water | 0.994  (0.986, 1.002) | **0.989**  **(0.978, 0.999)** | 1.000  (0.990, 1.011) |
| **Model 3** | Plain water | 1.000  (0.995, 1.005) | 1.001  (0.995, 1.007) | 0.999  (0.990, 1.008) |
|  | Beverage water | 1.002  (0.997, 1.007) | 1.004  (0.997, 1.011) | 1.000  (0.992, 1.008) |
|  | Food water | 0.995  (0.987, 1.003) | 0.990  (0.978, 1.001) | 1.000  (0.990, 1.011) |
|  |  | *Heart disease mortality risk* | | |
| **Model 1** | Plain water | 1.000  (0.994, 1.005) | 1.001  (0.994, 1.008) | 0.998  (0.988, 1.007) |
|  | Beverage water | 1.000  (0.995, 1.005) | 0.998  (0.991, 1.006) | 1.003  (0.995, 1.011) |
|  | Food water | 1.000  (0.992, 1.007) | 1.001  (0.990, 1.011) | 0.999  (0.987, 1.011) |
| **Model 2** | Plain water | 0.998  (0.993, 1.003) | 0.999  (0.992, 1.007) | 0.996  (0.986, 1.006) |
|  | Beverage water | 1.001  (0.996, 1.006) | 1.000  (0.993, 1.007) | 1.002  (0.993, 1.011) |
|  | Food water | 1.001  (0.994, 1.008) | 1.000  (0.991, 1.009) | 1.002  (0.991, 1.015) |
| **Model 3** | Plain water | 0.997  (0.991, 1.002) | 0.998  (0.991, 1.006) | 0.995  (0.985, 1.005) |
|  | Beverage water | 1.002  (0.997, 1.007) | 1.001  (0.994, 1.009) | 1.003  (0.994, 1.012) |
|  | Food water | 1.001  (0.994, 1.008) | 0.999  (0.991, 1.008) | 1.002  (0.990, 1.014) |

NHANES, National Health and Nutrition Examination Survey.

All estimates were calculated by multivariable Cox proportional hazards regression models, and results were expressed as hazard ratio (95% confidence interval). Total water intake is the sum of water intake from plain water, beverage and food. Covariates adjusted in model 1 included age, race, gender (excluded in men and women group), education, marital status, ratio of family income to poverty, body mass index. Covariates adjusted in model 2 included leisure time physical activity, dietary total energy, dietary protein, dietary carbohydrate, dietary total fat, dietary fiber, drinking status and smoking status in addition to those in model 1. Covariates adjusted in model 3 included cluster 1–4 of medical history in addition to those in model 2.

Boldness indicates a statistical significance.

Table S9. Associations of total water, plain water, beverage water and food water intake with all-cause mortality risk in the healthy NHANESs 1999–2014 participants.

| Water intake | 1^st^ quartile | 2^nd^ quartile | 3^rd^ quartile | 4^th^ quartile |
| --- | --- | --- | --- | --- |
| **Model 1** |  |  |  |  |
| Total water | 1.00 | **0.73 (0.54, 0.99)** | **0.67 (0.48, 0.94)** | **0.56 (0.37, 0.84)** |
| Plain water | 1.00 | 0.74 (0.52, 1.05) | **0.65 (0.47, 0.89)** | **0.70 (0.50, 0.99)** |
| Beverage water | 1.00 | 0.77 (0.58, 1.02) | 0.76 (0.54, 1.06) | 0.76 (0.56, 1.04) |
| Food water | 1.00 | 0.84 (0.62, 1.13) | **0.72 (0.52, 1.00)** | 0.72 (0.46, 1.13) |
| **Model 2** |  |  |  |  |
| Total water | 1.00 | 0.75 (0.54, 1.03) | 0.71 (0.48, 1.05) | **0.57 (0.36, 0.89)** |
| Plain water | 1.00 | 0.75 (0.52, 1.07) | **0.69 (0.49, 0.97)** | 0.73 (0.51, 1.05) |
| Beverage water | 1.00 | 0.78 (0.59, 1.03) | 0.78 (0.55, 1.12) | 0.78 (0.52, 1.16) |
| Food water | 1.00 | 0.89 (0.65, 1.22) | 0.76 (0.51, 1.11) | 0.75 (0.47, 1.19) |
| **Model 3** |  |  |  |  |
| Total water | - | - | - | - |
| Plain water | 1.00 | 0.77 (0.51, 1.16) | **0.70 (0.49, 1.00)** | 0.71 (0.48, 1.03) |
| Beverage water | 1.00 | **0.74 (0.56, 0.98)** | 0.73 (0.50, 1.07) | 0.70 (0.47, 1.05) |
| Food water | 1.00 | 0.88 (0.64, 1.20) | 0.78 (0.49, 1.22) | 0.81 (0.49, 1.33) |

NHANES, National Health and Nutrition Examination Survey.

All estimates were calculated by multivariable Cox proportional hazards regression models, and results were expressed as hazard ratio (95% confidence interval). Total water intake is the sum of water intake from plain water, beverage and food. Four kinds of water intake were categorized into four groups according to respective quartiles. Covariates adjusted in model 1 included age, race, gender, education, marital status, ratio of family income to poverty, body mass index. Covariates adjusted in model 2 included leisure time physical activity, dietary total energy, dietary protein, dietary carbohydrate, dietary total fat, dietary fiber, drinking status and smoking status in addition to those in model 1. Covariates adjusted in model 3 included the other kinds of water source in addition to those in model 2. That is, for plain water, we included beverage water and food water as added covariates; for beverage water, we included plain water and food water as added covariates; and for food water, we included plain water and beverage water as added covariates.

The range of quartiles for total water intake were < 2184 (Q1), 2184–3042 (Q2), 3043–4123 (Q3) and 4124–8485 g/day (Q4). The range of quartiles for plain water intake were < 296 (Q1), 296–770 (Q2), 771–1416 (Q3) and 1417–3776 g/day (Q4). The range of quartiles for beverage water intake were < 705 (Q1), 705–1076 (Q2), 1077–1584 (Q3) and 1585–3464 g/day (Q4). The range of quartiles for food water intake were < 517 (Q1), 517–912 (Q2), 913–1624 (Q3) and 1625–3784 g/day (Q4).

Boldness indicates a statistical significance.

Table S10. Associations of total water, plain water, beverage water and food water intake with malignant neoplasms/cancer mortality risk in the NHANESs 1999–2014 participants without cancer history.

| Water intake | 1^st^ quartile | 2^nd^ quartile | 3^rd^ quartile | 4^th^ quartile |
| --- | --- | --- | --- | --- |
| **Model 1** |  |  |  |  |
| Total water | 1.00 | 0.91 (0.69, 1.18) | 0.75 (0.55, 1.01) | 0.78 (0.58, 1.05) |
| Plain water | 1.00 | 0.87 (0.64, 1.18) | **0.74 (0.55, 1.00)** | 0.78 (0.58, 1.05) |
| Beverage water | 1.00 | 0.79 (0.57, 1.11) | 0.74 (0.52, 1.06) | 0.95 (0.66, 1.35) |
| Food water | 1.00 | 0.98 (0.73, 1.32) | 0.79 (0.55, 1.15) | **0.64 (0.43, 0.93)** |
| **Model 2** |  |  |  |  |
| Total water | 1.00 | 0.97 (0.74, 1.27) | 0.83 (0.60, 1.15) | 0.90 (0.64, 1.25) |
| Plain water | 1.00 | 0.90 (0.66, 1.25) | 0.80 (0.59, 1.09) | 0.83 (0.60, 1.15) |
| Beverage water | 1.00 | 0.81 (0.57, 1.13) | 0.75 (0.52, 1.09) | 0.97 (0.67, 1.40) |
| Food water | 1.00 | 1.17 (0.84, 1.62) | 0.98 (0.66, 1.46) | 0.80 (0.53, 1.20) |
| **Model 3** |  |  |  |  |
| Total water | 1.00 | 0.97 (0.74, 1.28) | 0.83 (0.60, 1.16) | 0.90 (0.64, 1.26) |
| Plain water | 1.00 | 0.90 (0.65, 1.25) | 0.80 (0.59, 1.09) | 0.83 (0.60, 1.15) |
| Beverage water | 1.00 | 0.81 (0.57, 1.14) | 0.75 (0.52, 1.09) | 0.97 (0.67, 1.40) |
| Food water | 1.00 | 1.17 (0.85, 1.63) | 0.99 (0.67, 1.49) | 0.81 (0.54, 1.21) |
| **Model 4** |  |  |  |  |
| Total water | - | - | - | - |
| Plain water | 1.00 | 0.92 (0.64, 1.31) | 0.84 (0.61, 1.17) | 0.90 (0.63, 1.28) |
| Beverage water | 1.00 | 0.79 (0.56, 1.12) | 0.73 (0.50, 1.06) | 0.93 (0.64, 1.35) |
| Food water | 1.00 | 1.18 (0.85, 1.65) | 1.03 (0.67, 1.60) | 0.85 (0.56, 1.30) |

NHANES, National Health and Nutrition Examination Survey.

All estimates were calculated by multivariable Cox proportional hazards regression models, and results were expressed as hazard ratio (95% confidence interval). Total water intake is the sum of water intake from plain water, beverage and food. Four kinds of water intake were categorized into four groups according to respective quartiles. Covariates adjusted in model 1 included age, race, gender, education, marital status, ratio of family income to poverty, body mass index. Covariates adjusted in model 2 included leisure time physical activity, dietary total energy, dietary protein, dietary carbohydrate, dietary total fat, dietary fiber, drinking status and smoking status in addition to those in model 1. Covariates adjusted in model 3 included cluster 1–4 of medical history in addition to those in model 2. But cluster 4 of medical history did not include the medical history of cancer. Covariates adjusted in model 4 included the other kinds of water source in addition to those in model 3. That is, for plain water, we included beverage water and food water as added covariates; for beverage water, we included plain water and food water as added covariates; and for food water, we included plain water and beverage water as added covariates.

The range of quartiles for total water intake were < 2155 (Q1), 2155–2984 (Q2), 2985–4067 (Q3) and 4068–8516 g/day (Q4). The range of quartiles for plain water intake were < 311 (Q1), 311–755 (Q2), 756–1391 (Q3) and 1392–3776 g/day (Q4). The range of quartiles for beverage water intake were < 677 (Q1), 677–1037 (Q2), 1038–1532 (Q3) and 1533–3464 g/day (Q4). The range of quartiles for food water intake were < 530 (Q1), 531–924 (Q2), 925–1620 (Q3) and 1621–3802 g/day (Q4).

Boldness indicates a statistical significance.

Table S11. Associations of total water, plain water, beverage water and food water intake with heart disease mortality risk in the NHANESs 1999–2014 participants without cardiovascular disease history.

| Water intake | 1^st^ quartile | 2^nd^ quartile | 3^rd^ quartile | 4^th^ quartile |
| --- | --- | --- | --- | --- |
| **Model 1** |  |  |  |  |
| Total water | 1.00 | **0.67 (0.46, 0.97)** | **0.67 (0.46, 0.98)** | 0.68 (0.42, 1.10) |
| Plain water | 1.00 | 0.97 (0.70, 1.35) | 0.76 (0.51, 1.13) | 0.90 (0.62, 1.31) |
| Beverage water | 1.00 | 0.89 (0.60, 1.30) | 0.73 (0.50, 1.08) | **0.54 (0.37, 0.78)** |
| Food water | 1.00 | 1.22 (0.85, 1.76) | 0.82 (0.56, 1.19) | 1.01 (0.58, 1.74) |
| **Model 2** |  |  |  |  |
| Total water | 1.00 | 0.74 (0.50, 1.11) | 0.79 (0.52, 1.21) | 0.85 (0.48, 1.49) |
| Plain water | 1.00 | 1.03 (0.73, 1.45) | 0.77 (0.50, 1.18) | 0.95 (0.65, 1.39) |
| Beverage water | 1.00 | 0.97 (0.66, 1.42) | 0.85 (0.57, 1.25) | 0.70 (0.46, 1.06) |
| Food water | 1.00 | 1.38 (0.93, 2.05) | 0.96 (0.65, 1.44) | 1.16 (0.64, 2.10) |
| **Model 3** |  |  |  |  |
| Total water | 1.00 | 0.75 (0.50, 1.11) | 0.79 (0.52, 1.19) | 0.83 (0.47, 1.45) |
| Plain water | 1.00 | 1.02 (0.73, 1.44) | 0.77 (0.50, 1.17) | 0.94 (0.64, 1.38) |
| Beverage water | 1.00 | 0.97 (0.66, 1.42) | 0.84 (0.57, 1.25) | 0.69 (0.45, 1.06) |
| Food water | 1.00 | 1.36 (0.91, 2.03) | 0.94 (0.64, 1.40) | 1.12 (0.63, 2.02) |
| **Model 4** |  |  |  |  |
| Total water | - | - | - | - |
| Plain water | 1.00 | 1.03 (0.72, 1.48) | 0.77 (0.51, 1.16) | 0.91 (0.59, 1.39) |
| Beverage water | 1.00 | 0.95 (0.65, 1.40) | 0.82 (0.55, 1.22) | 0.66 (0.42, 1.05) |
| Food water | 1.00 | 1.31 (0.87, 1.97) | 0.90 (0.59, 1.37) | 1.14 (0.63, 2.09) |

NHANES, National Health and Nutrition Examination Survey.

All estimates were calculated by multivariable Cox proportional hazards regression models, and results were expressed as hazard ratio (95% confidence interval). Total water intake is the sum of water intake from plain water, beverage and food. Four kinds of water intake were categorized into four groups according to respective quartiles. Covariates adjusted in model 1 included age, race, gender, education, marital status, ratio of family income to poverty, body mass index. Covariates adjusted in model 2 included leisure time physical activity, dietary total energy, dietary protein, dietary carbohydrate, dietary total fat, dietary fiber, drinking status and smoking status in addition to those in model 1. Covariates adjusted in model 3 included cluster 1–4 of medical history in addition to those in model 2. Covariates adjusted in model 4 included the other kinds of water source in addition to those in model 3. That is, for plain water intake, we included beverage water intake and food water intake as added covariates; for beverage water intake, we included plain water intake and food water intake as added covariates; and for food water intake, we included plain water intake and beverage water intake as added covariates.

The range of quartiles for total water intake were < 2182 (Q1), 2182–3014 (Q2), 3015–4101 (Q3) and 4102–8516 g/day (Q4). The range of quartiles for plain water intake were < 315 (Q1), 315–770 (Q2), 771–1403 (Q3) and 1404–3776 g/day (Q4). The range of quartiles for beverage water intake were < 685 (Q1), 685–1044 (Q2), 1045–1538 (Q3) and 1539–3464 g/day (Q4). The range of quartiles for food water intake were < 537 (Q1), 537–942 (Q2), 943–1641 (Q3) and 1642–3802 g/day (Q4).

Boldness indicates a statistical significance.
